# Beyond Conventional: A Review of Phytochemical‐Hydrogel Systems Enhanced by AI and 3D Printing for Chronic Wound Management

**DOI:** 10.1002/advs.77770

**Published:** 2026-09-21

**Authors:** Yitao Zhou, Xing Liu, Cuiyi Wang, Xiaoxuan Zhao, Pengrun Zhang, Jingjing Gu, Qiuhua Sun, Malcolm Xing, Yeqin Yang

**Affiliations:** ^1^ School of Nursing Zhejiang Chinese Medical University Hangzhou Zhejiang China; ^2^ College of Basic Medical Sciences Zhejiang Chinese Medical University Hangzhou Zhejiang China; ^3^ Department of Mechanical Engineering University of Manitoba Winnipeg Manitoba Canada

**Keywords:** 3D bioprinting, artificial intelligence, chronic wounds, phytochemical hydrogels, stimuli‐responsive materials

## Abstract

Chronic wounds represent a global healthcare challenge, characterized by persistent inflammation, microbial infection, and compromised tissue regeneration, often refractory to conventional therapies. Natural phytochemicals, with their inherent diverse bioactivities, provide a multi‐target approach to modulate inflammation and accelerate tissue repair, overcoming the limitations of single‐agent interventions. However, their clinical utility is frequently limited by poor physicochemical stability, low bioavailability, and insufficient retention at the wound site. Advanced hydrogel platforms serve as effective delivery systems, capable of encapsulating these potent compounds to ensure localized, sustained, and on‐demand release, addressing critical translational barriers. This review examines the wound‐healing mechanisms and molecular targets of key phytochemical classes, critically analyzing their physicochemical limitations. We then discuss the rational design principles of phytochemical hydrogel systems, encompassing stimuli‐responsive networks, externally triggered release modalities, and 3D‐printed scaffolds for precise spatial drug distribution. We also highlight the emerging role of artificial intelligence in accelerating this field, from predicting hydrogel characteristics and screening novel phytochemicals to optimizing formulation strategies. Finally, we propose a strategic translational roadmap, addressing crucial aspects such as botanical standardization, scalable manufacturing, and regulatory pathways, to support the clinical translation of these systems.

## Graphical abstract

This review systematically bridges chronic wound pathology with natural phytochemical hydrogel therapeutics. A pathology‐to‐phytochemical mechanistic mapping framework is established, linking specific wound hallmarks to targeted phytochemical interventions. Stimuli‐responsive release strategies, 3D‐printed architectures, and emerging artificial intelligence‐driven design paradigms are critically evaluated. Current clinical evidence and translational barriers are discussed, providing a roadmap for next‐generation intelligent phytochemical hydrogel wound dressings.

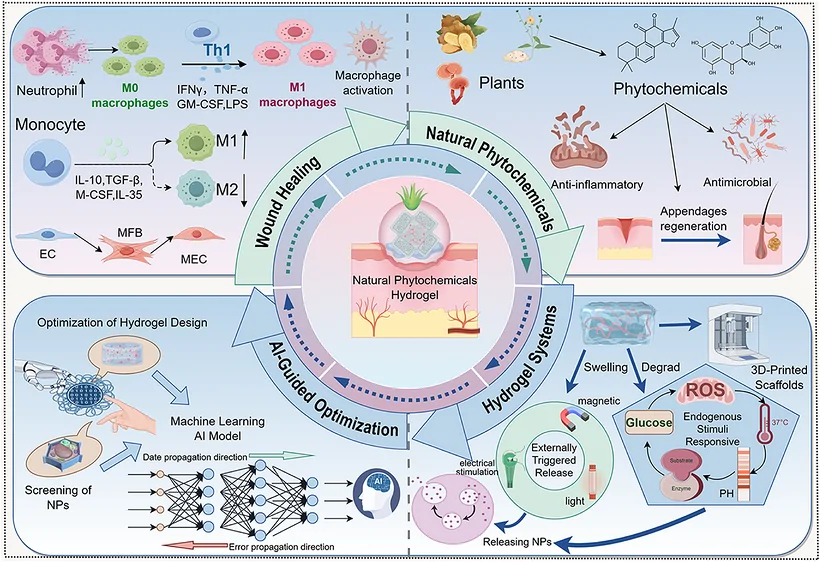

## 1 Introduction

Chronic wounds, such as diabetic ulcers and pressure injuries, remain among the leading causes of long‐term disability worldwide, placing a heavy burden on patients and healthcare systems [1, 2]. Despite advances in wound care, the current challenge lies in developing effective, economical, and practical novel wound healing materials and tracking their progression. The fusion of natural phytochemicals with hydrogel biomaterials has attracted growing attention in regenerative wound care, supporting localized delivery, sustained release, and active modulation of healing [3, 4]. Traditionally, wound management has relied on passive dressings that merely protect the wound surface. Recent advances in phytochemical encapsulation and hydrogel engineering now offer multifunctional systems capable of actively regulating inflammation, infection, and tissue regeneration [3, 4]. Several recent studies have advanced our understanding of individual aspects of this landscape. The design and performance of natural‐polymer hydrogels based on chitosan, alginate, or hyaluronic acid as wound dressings have been comprehensively surveyed [5, 6]. Independently, the wound‐relevant pharmacology of plant secondary metabolites has been cataloged [7, 8], and the specific role of traditional Chinese medicine compounds in hydrogel networks for diabetic ulcer repair has been examined [9]. Stimuli‐responsive and programmable hydrogels have likewise been reviewed in broad biomedical contexts [10, 11]. What remains absent, however, is a framework that systematically links the physicochemical bottlenecks of each phytochemical class to the rational design logic of their hydrogel carriers, while simultaneously incorporating AI‐driven formulation discovery and 3D‐printed spatial control as enabling tools within a single translational pipeline. This review sets out to fill that gap. Section 2 provides a concise account of chronic wound pathobiology, establishing the cellular and molecular targets that phytochemical hydrogel systems must engage. Section 3 classifies the major phytochemical families, polyphenols, terpenoids, alkaloids, and quinones, by their wound‐relevant mechanisms and, importantly, identifies the specific physicochemical barriers (hydrophobicity, oxidative lability, dose‐dependent toxicity) that have limited each class in practice. Section 4 then presents the hydrogel design strategies chosen to overcome those barriers: endogenous stimuli‐responsive networks, externally triggered release modalities, and 3D‐printed architectures for spatially graded delivery. Section 5 evaluates how AI is beginning to accelerate both hydrogel property prediction and phytochemical candidate screening. Section 6 assesses available clinical evidence and maps a translational roadmap covering botanical standardization, scalable manufacture, and regulatory pathways (Figure 1).

**FIGURE 1:**
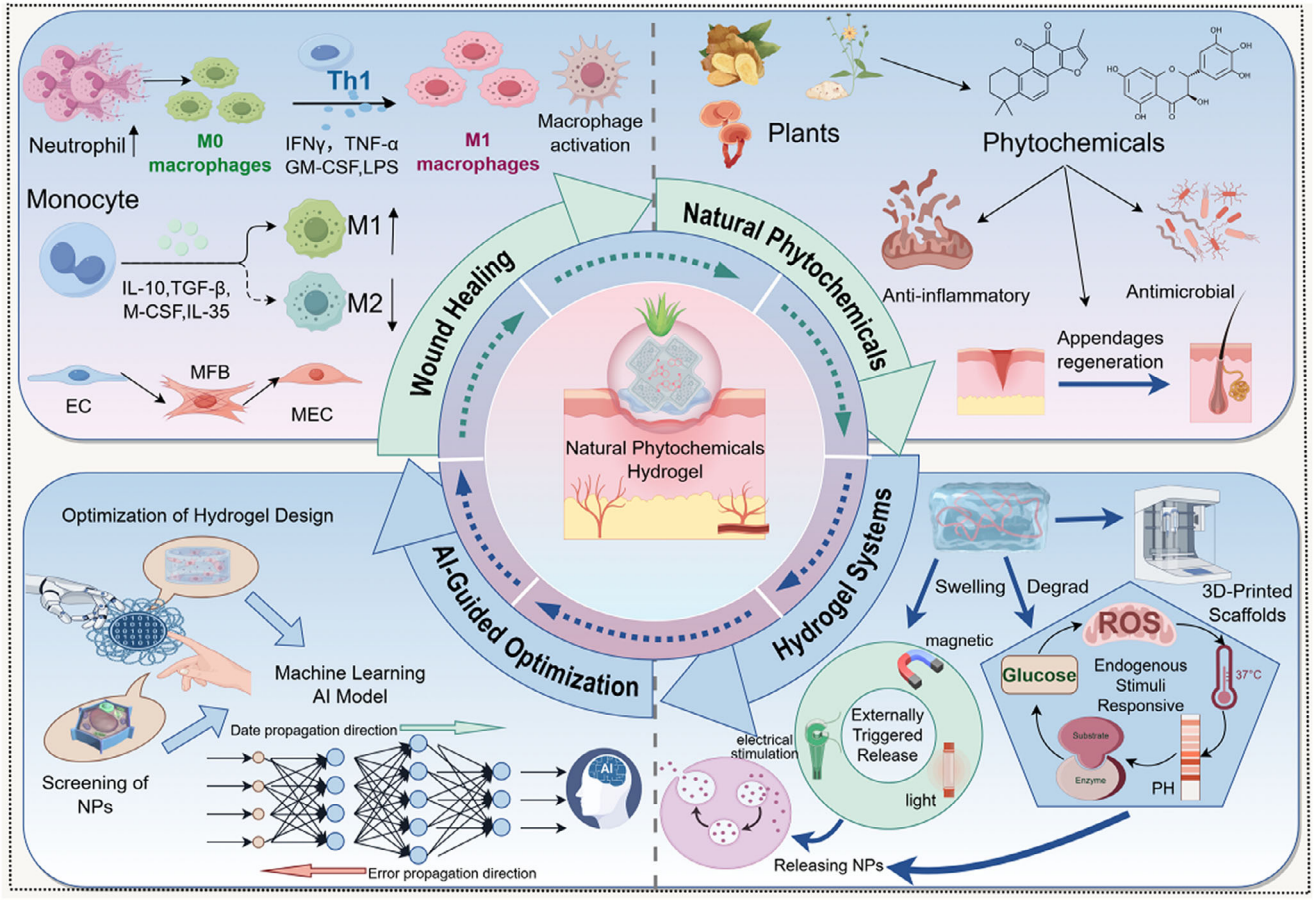
Schematic overview of natural phytochemical hydrogels for wound healing.

## 2 Wound Healing Mechanisms

Wound healing is a complex and dynamic process that consists of a series of temporally ordered but partially overlapping biological events. These events work together to restore tissue structure and function. Classically, wound repair is divided into four main stages: hemostasis, inflammation, proliferation, and remodeling [12]. The overall progression of these four stages and the key cellular contributors are illustrated in Figure 2A [12], while the sequential events of wound closure, ranging from clot formation to late scar remodeling, are depicted in greater detail in Figure 2B [13]. Chronic wounds, often referred to as difficult to heal lesions, are characterized by impaired tissue restoration and a failure to follow the normal sequence of repair. The characteristic cellular and molecular disturbances observed in chronic ulcers, including epidermal hyperproliferation, persistent inflammation, dysfunctional leukocytes, fibroblast senescence, and reduced angiogenesis, are visualized in Figure 2C [14]. Any wound has the potential to progress into a chronic wound, most commonly in the form of pressure, diabetic, venous, or arterial ulcers [6].

**FIGURE 2:**
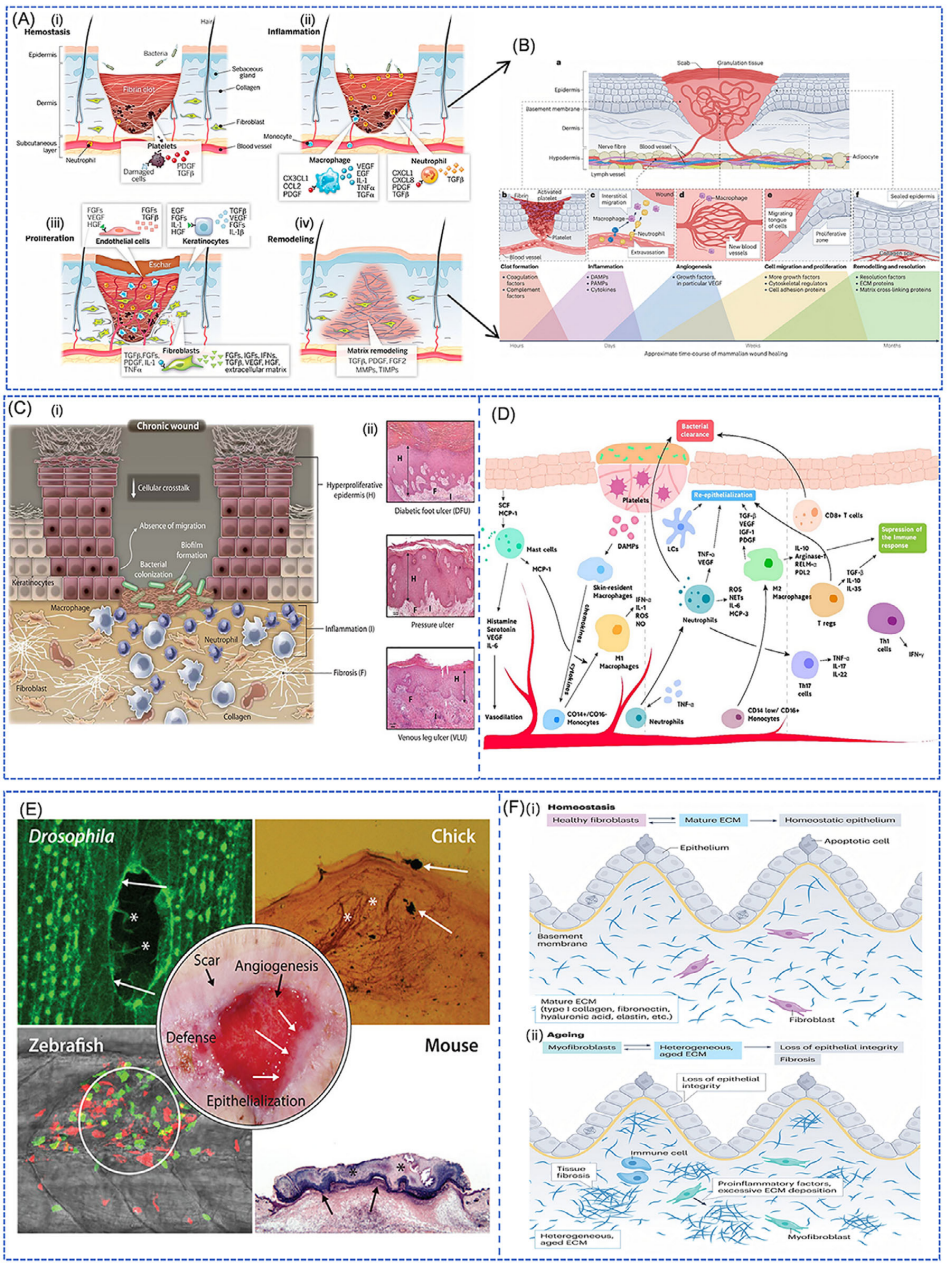
Integrated visualization of wound healing mechanisms and wound pathology. A) Classical four‐stage wound healing process. Reproduced with permission [12]. Copyright 2014, American Association for the Advancement of Science. B) Expanded schematic of wound closure showing clot formation, immune activation, granulation tissue development, re‐epithelialization, and late ECM remodeling. Reproduced with permission [13]. Copyright 2024, Springer Nature. C) Pathological features of chronic wounds. Reproduced with permission [14]. Copyright 2014, American Association for the Advancement of Science. D) Overview of immune orchestration during acute wound healing. Reproduced under the terms of the CC‐BY license [15]. Copyright 2021, The Authors, published by MDPI. E) Conserved mechanisms of repair across model organisms demonstrating shared processes such as leukocyte recruitment, angiogenesis, epithelial migration, innervation, and scar formation. Reproduced with permission [14]. Copyright 2014, American Association for the Advancement of Science. F) Role of ECM mechanics in wound repair. Reproduced with permission [16]. Copyright 2023, Springer Nature.

### 2.1 Hemostasis

The hemostatic response begins with platelet adhesion and activation, simultaneously triggering the coagulation cascade [17]. The resulting fibrin clot forms a provisional matrix essential for subsequent healing phases [18, 19]. Activated platelets secrete over 300 proteins from their granules, including growth factors and chemokines that initiate inflammatory cell recruitment and tissue repair signaling [20, 21]. This hemostatic plug acts not only to control hemorrhage immediately but also as a scaffold for migrating cells and a reservoir of bioactive molecules that guides the transition to the inflammatory phase.

### 2.2 Inflammation

The inflammatory phase is characterized by precisely orchestrated immune cell recruitment, initiating with neutrophil infiltration within hours and subsequent monocyte arrival [22]. Neutrophils play roles beyond antimicrobial defense by secreting matrix metalloproteinases (MMPs) and regulatory molecules that regulate the balance between tissue damage and repair initiation [23]. The critical shift from pro‐inflammatory M1 to anti‐inflammatory M2 macrophage phenotypes shapes healing outcomes, as M2 polarization supports tissue repair via growth factor secretion and debris clearance [24]. The coordinated recruitment and functional specialization of neutrophils, monocytes, macrophages, mast cells, and various T‐cell subsets during this phase are summarized in Figure 2D, highlighting their roles in bacterial clearance, cytokine signaling, and inflammatory resolution [15]. Nuclear factor‐κB (NF‐κB) signaling acts as a master regulator of inflammatory gene expression, orchestrating cytokine production and immune cell activation [25]. Successful resolution of inflammation depends on tight temporal regulation: prolonged inflammatory responses are a hallmark of chronic wounds, whereas premature resolution may result in infection and healing failure.

### 2.3 Proliferation

The proliferative phase is characterized by coordinated angiogenesis, fibroblast activation, and re‐epithelialization, all driven by hypoxia and growth factor signaling [26]. Angiogenesis is mediated by endothelial cell sprouting and vessel maturation, which re‐establishes blood supply to support tissue regeneration [27]. Concurrently, fibroblast proliferation and myofibroblast differentiation support collagen synthesis and wound contraction [28, 29], while reciprocal keratinocyte‐fibroblast paracrine signaling further coordinates epidermal and dermal repair processes [28, 29]. Hypoxia‐inducible factor‐1α (HIF‐1α) acts as a master regulator, directly controlling VEGF expression and orchestrating angiogenic responses to tissue oxygen demands [30]. Granulation tissue forms during this phase, creating a vascularized, growth factor‐rich extracellular matrix (ECM) scaffold that sustains cellular migration and proliferation. The mechanical properties of the newly formed ECM, especially its stiffness and viscoelasticity, are central to maintaining fibroblast activity and epithelial regeneration, consistent with the homeostatic ECM organization shown in Figure 2F‐i [16].

### 2.4 Remodeling

The remodeling phase extends for months to years, characterized by collagen reorganization and matrix maturation that gradually enhance tissue tensile strength [31]. More generally, evidence from fibrotic tissue remodeling shows that the balance between MMPs and their inhibitors regulates ECM turnover [32]. Successful remodeling depends on myofibroblast apoptosis and removal of excess collagen, thereby preventing pathological scarring and restoring functional tissue integrity [33]. Disruption of this finely balanced remodeling process leads to heterogeneous ECM architecture and persistent myofibroblast activation, which ultimately impair epithelial integrity and tissue function, as illustrated in Figure 2F(ii) [16]. Notably, these core repair mechanisms are broadly conserved across species, from invertebrates to mammals, Figure 2E [14]. Given the multifactorial nature of chronic wound pathology, the following section examines how natural phytochemicals can simultaneously engage multiple therapeutic targets.

## 3 Natural Phytochemicals in Wound Healing

Natural phytochemicals play a valuable role in wound healing because they can regulate several biological processes simultaneously, including inflammation, oxidative stress, microbial control, and tissue regeneration, Figure 3C,G [7, 34]. Their diverse chemical structures allow them to act on multiple signaling pathways, offering broader therapeutic effects than single‐target drugs, Figure 3A,F [7, 8]. In addition, botanical source selection and extraction‐related variables can substantially shape phytochemical composition and antioxidant profiles, contributing to inter‐study variability in reported effects, Figure 3B [35]. However, many phytochemicals have poor water solubility, limited stability, and low bioavailability, which restrict their direct clinical application; representative bioactive structures and target‐level mechanisms further illustrate why formulation and delivery are often rate‐limiting for clinical translation, Figure 3D,E [36, 37]. These limitations highlight the need for suitable delivery systems that can stabilize phytochemicals and enable controlled, effective release at the wound site [4, 38]. Hydrogels, with their hydrated three‐dimensional polymer networks and tunable physicochemical properties, provide an ideal platform to encapsulate and protect phytochemicals while enabling sustained, localized, and bioactive delivery at the wound site. Importantly, the therapeutic relevance of these phytochemicals can only be fully appreciated when mapped against the specific pathological axes of chronic wounds established in Section 2. Chronic wounds are driven by interlinked hallmarks: (i) persistent NF‐κB‐driven inflammation, (ii) excessive ROS accumulation, (iii) bacterial colonization and biofilm formation, (iv) deficient VEGF/HIF‐1α‐mediated angiogenesis, and (v) M1‐to‐M2 macrophage polarization imbalance. Rather than acting on a single target, most phytochemicals simultaneously engage two or more of these axes, which constitutes their principal advantage over conventional single‐agent therapies. To provide a consolidated overview, Table 1 maps representative phytochemicals from each major class to the specific chronic wound hallmarks they address, the key molecular targets involved, and the supporting evidence. This pathology‐to‐phytochemical mapping is intended to guide the reader through the mechanistic rationale underpinning the hydrogel design strategies discussed in Section 4. Based on their structural features and biological activities, the major classes of phytochemicals relevant to wound healing are summarized in Figure 3.

**FIGURE 3:**
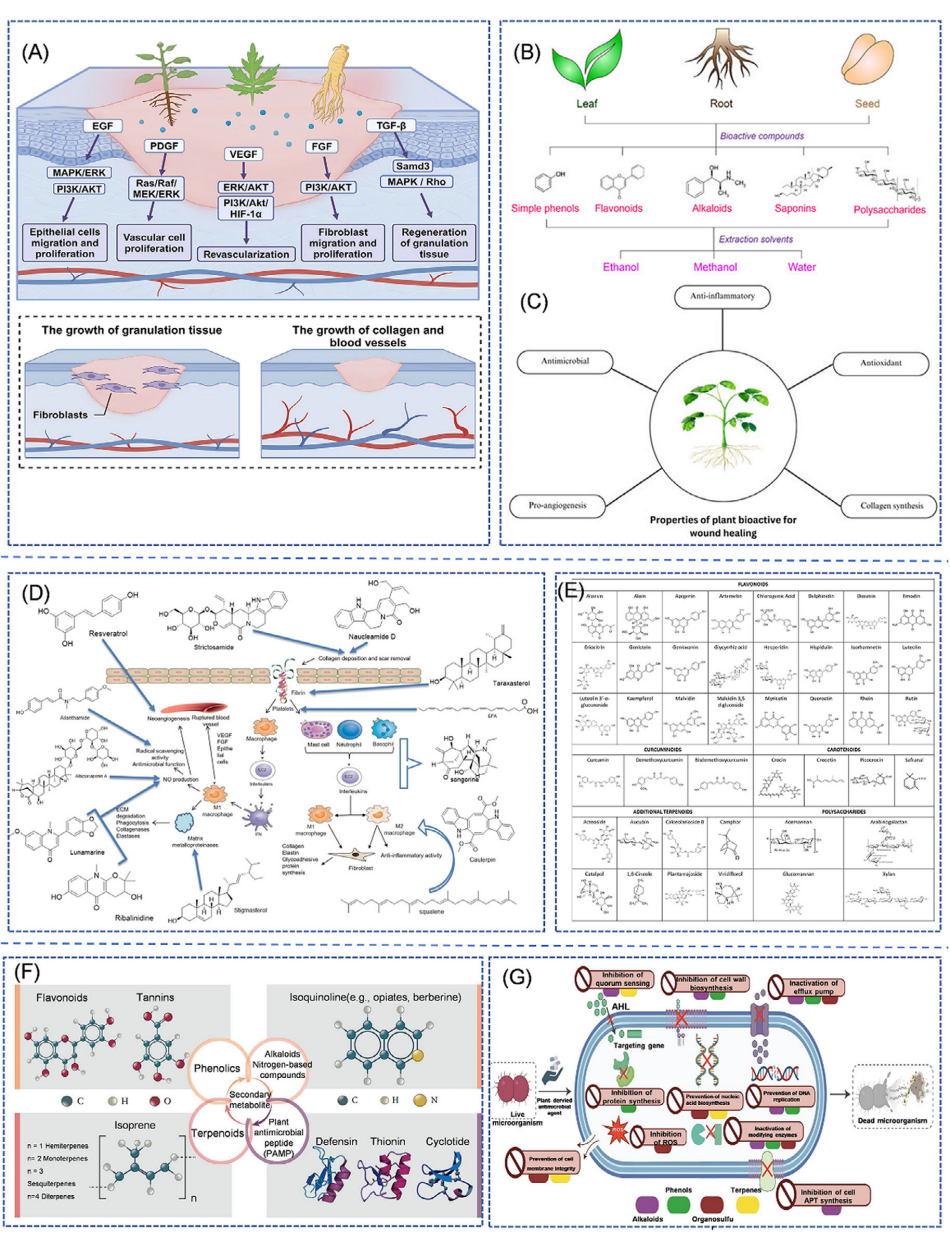
Overview of phytochemical classes, structures, and biological pathways involved in wound healing. A) Key wound‐healing signaling pathways modulated by phytochemicals, including inflammatory and antioxidant signaling. Reproduced under the terms of the CC‐BY 4.0 license [8]. Copyright 2025, The Authors, published by Wiley‐VCH GmbH. B) Botanical sources and extraction‐related features shaping antioxidant phytochemical composition. Reproduced under the terms of the CC‐BY 4.0 license [35]. Copyright 2023, The Authors, published by MDPI. C) Core biofunctions promoting re‐epithelialization, angiogenesis, and extracellular matrix remodeling. Reproduced under the terms of the CC‐BY 4.0 license [34]. Copyright 2025, The Authors, published by MDPI. D) Mechanistic schematic showing how selected natural compounds act on specific targets during wound healing. Reproduced with permission [36]. Copyright 2024, Wiley‐VHCA AG. E) Representative primary bioactive phytochemical structures implicated in wound repair. Reproduced under the terms of the CC‐BY 4.0 license [37]. Copyright 2022, The Authors, published by MDPI. F) Structural classification of principal phytochemical groups, including polyphenols, terpenoids, alkaloids, quinones, polysaccharides, and peptides. Reproduced under the terms of the CC‐BY 4.0 license [7]. Copyright 2025, The Authors, published by John Wiley & Sons Ltd. G) Proposed antimicrobial mechanisms of plant‐derived compounds. Reproduced under the terms of the CC‐BY 4.0 license [7]. Copyright 2025, The Authors, published by John Wiley & Sons Ltd.

**TABLE 1:** Pathology‐to‐Phytochemical Mechanistic Mapping: Representative Phytochemicals Targeting Chronic Wound Pathological Hallmarks.

Chronic wound pathological hallmark	Key molecular targets/pathways	Representative phytochemical	Class	Mechanistic action	Refs.
Persistent inflammation	NF‐κB; TNF‐α; IL‐6; COX‐2	Curcumin	Nonflavonoid polyphenol	Suppresses NF‐κB signaling; downregulates TNF‐α and IL‐6; inhibits fibrosis via TGF‐β/Smad modulation	[39, 40]
	MAPK (ERK1/2, JNK, p38)	Quercetin	Flavonoid	Attenuates NF‐κB and JNK activation; inhibits phosphorylation of JNK (ERK1/2 and p38 phosphorylation unaffected)	[41]
	MRSA virulence factors; NF‐κB	Baicalin+Sanguinarine	Flavonoid+Alkaloid	Carrier‐free co‐assembly suppresses MRSA virulence factors; mitigates virulence‐driven inflammation	[42]
Excessive oxidative stress	Nrf2/HO‐1; ROS	EGCG	Flavonoid	EGCG‐mediated ROS‐cleavable borate‐ester bonds regulate release from a QOF‐EGCG/Hb@PDA hydrogel; Hb@PDA nanoparticles serve as the oxygen‐delivery component	[43]
	ROS; therapeutic window	Shikonin	Quinone	Fe/Shikonin nanoparticles provide NIR‐triggered photothermal antibacterial activity and ROS scavenging in a wet‐adhesive patch	[44]
Bacterial infection and biofilm	Bacterial membrane; EPS synthesis	Sanguinarine	Alkaloid	Disrupts *S. pseudintermedius* biofilms; inhibits EPS synthesis, weakening biofilm structural integrity	[45]
	MRSA; MAPK; AMPK	Berberine	Alkaloid	Reduces drug‐resistant bacterial load; modulates host MAPK and AMPK signaling to enhance granulation tissue	[46]
	Microbial RNA/protein synthesis; TNF‐α; IL‐6	Terpenoids (general)	Terpenoid	Suppress microbial RNA and protein synthesis; reduce TNF‐α and IL‐6 in mast cells	[47]
Impaired angiogenesis	VEGF	Asiaticoside	Terpenoid	Promotes angiogenesis via VEGF upregulation; dual‐crosslinked hydrogel enhances stability in diabetic foot ulcers	[48, 49]
	SRC/MEK/ERK	Total alkaloids (*Leonurus japonicus*)	Alkaloid	Enhances proliferation, collagen deposition, and angiogenesis through SRC/MEK/ERK signaling	[50]
Macrophage polarization imbalance	M1/M2; tissue hypoxia	EGCG	Flavonoid	Hb@PDA‐mediated oxygen delivery alleviates hypoxia; pH/ROS‐dual‐responsive release drives M2 macrophage polarization	[43]
	M1/M2 rebalancing; neovascularization	Matrine	Alkaloid	Rebalances macrophage phenotypes (M1 to M2) when co‐delivered with MSC‐derived exosomes in self‐healing hydrogel	[51]
	NF‐κB; angiogenesis	Quercetin	Flavonoid	Modulates NF‐κB‐mediated inflammation; promotes barrier restoration and collagen remodeling	[52]

Abbreviations: Akt, protein kinase B; AMPK, AMP‐activated protein kinase; COX‐2, cyclooxygenase‐2; ECM, extracellular matrix; EGCG, epigallocatechin gallate; EPS, extracellular polymeric substance; ERK, extracellular signal‐regulated kinase; HIF‐1α, hypoxia‐inducible factor 1 alpha; HO‐1, heme oxygenase‐1; IL‐6, interleukin‐6; JNK, c‐Jun N‐terminal kinase; MAPK, mitogen‐activated protein kinase; MEK, MAPK/ERK kinase; MMP, matrix metalloproteinase; MRSA, methicillin‐resistant Staphylococcus aureus; MSC, mesenchymal stem cell;NF‐κB, nuclear factor kappa‐light‐chain‐enhancer of activated B cells; Nrf2, nuclear factor erythroid 2‐related factor 2; PI3K, phosphoinositide 3‐kinase; ROS, reactive oxygen species; SRC, proto‐oncogene tyrosine‐protein kinase Src; TGF‐β, transforming growth factor beta; TNF‐α, tumor necrosis factor alpha; VEGF, vascular endothelial growth factor.

### 3.1 Classification and Bioactivity of Phytochemicals

Natural phytochemicals are systematically classified based on their chemical structures and primary bioactivities, both of which are crucial for their therapeutic effectiveness in wound healing. This section reviews the roles of major phytochemical classes such as polyphenols, terpenoids, alkaloids, and quinones in wound healing.

#### 3.1.1 Polyphenols

Dietary polyphenols are generally divided into two primary categories: flavonoid and nonflavonoid types, Figure 4A [8]. A wide range of polyphenolic substances with a benzo‐γ‐pyrone configuration, known as flavonoids, are found universally in plants [53]. These compounds are produced through the phenylpropanoid pathway, and current studies suggest that phenolic secondary metabolites, especially flavonoids, play a key role in mediating a wide range of pharmacological effects [53]. Flavonoids possess a characteristic chemical structure comprising a fifteen‐carbon framework, featuring two benzene rings connected by a central heterocyclic pyran ring [53]. This structural scaffold gives rise to multiple flavonoid subclasses with distinct substitution patterns and bioactivities, which are schematically summarized with representative structures and source examples in Figure 4B [9]. They are potent modulators of inflammation and oxidative stress and exert five major therapeutic effects relevant to wound repair, Figure 4C [54]. Flavonoids encompass nearly 10,000 structurally related compounds that share the characteristic C6─C3─C6 backbone [55]. In recent years, these secondary metabolites have garnered exponential interest due to their purported health benefits. Numerous studies have reported that flavonoids and other phenolic compounds exhibit a broad spectrum of bioactivities, including antimicrobial, antioxidant, antiviral, anti‐inflammatory, antihyperlipidemic, and antidiabetic effects, as well as neuroprotective, hepatoprotective, and cardioprotective properties [55, 56, 57]. Previous studies have reported that many flavonoids exert antioxidant effects by scavenging free radicals and preventing their formation. Mechanistically, several flavonoids have been shown to upregulate VEGF and MMP‐9 expression, which can in turn suppress pro‐inflammatory IL‐6 and TNF‐α while inducing anti‐inflammatory IL‐10. They improve the antioxidant defense system, primarily during the inflammatory and proliferative phases of healing, Figure 4D [54, 58].

**FIGURE 4:**
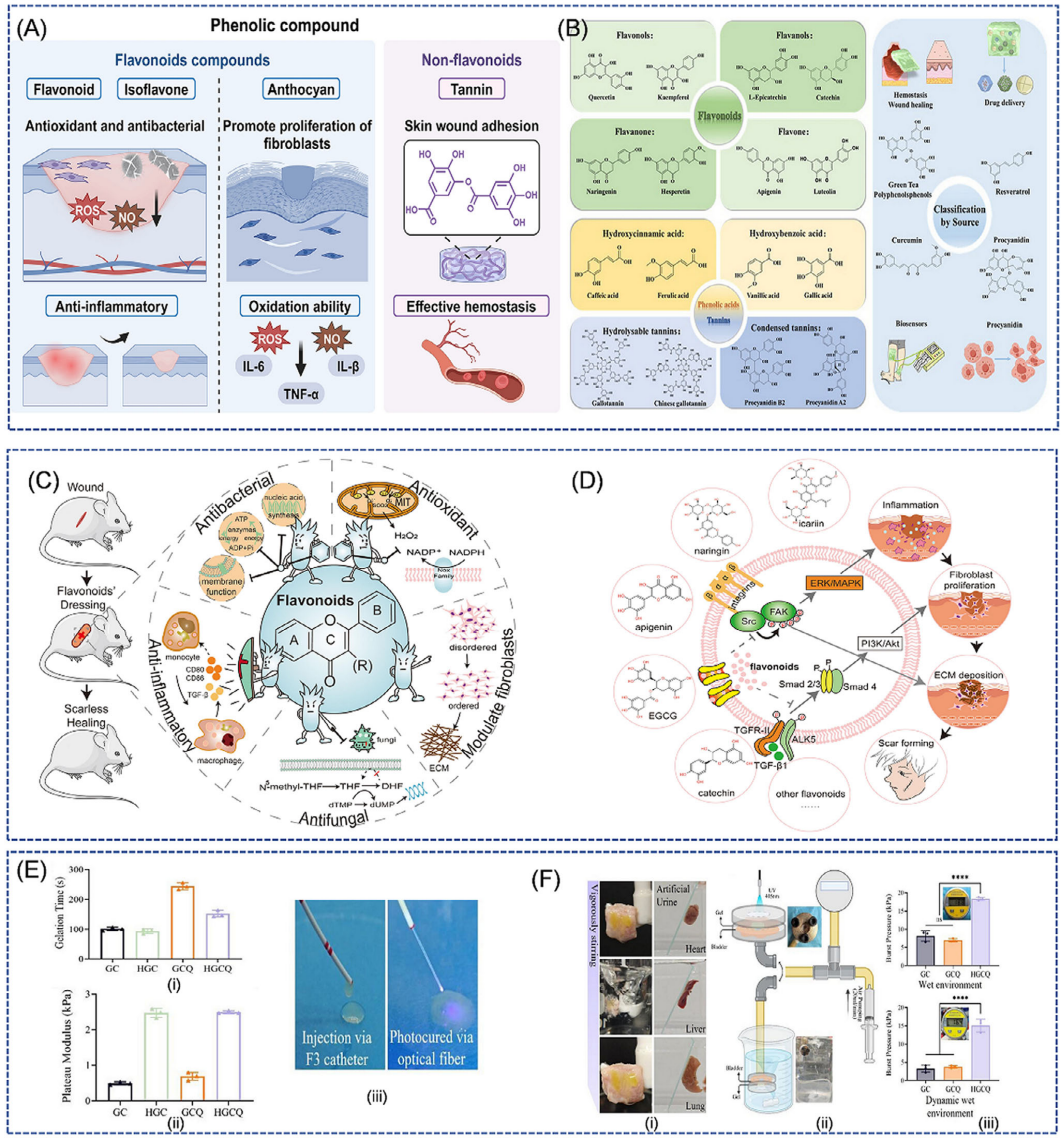
Biological roles of phenolic compounds, key wound healing effects of flavonoids. A) Polyphenols include two major categories: flavonoids and non‐flavonoids. Reproduced with permission [8]. Copyright 2025, Wiley‐VCH GmbH. B) Classification and representative chemical structures of major flavonoid subclasses and selected plant source polyphenols. Reproduced with permission [9]. Copyright 2025, Wiley‐VCH GmbH. C) The five major biological effects of flavonoid‐laden dressings relevant to wound repair. Reproduced under the terms of the CC‐BY 4.0 license [54]. Copyright 2022, The Authors, published by Frontiers. D) Flavonoids promote scar‐free regeneration by regulating multiple signaling pathways involved in inflammation, oxidative stress, angiogenesis, and matrix remodeling. Reproduced under the terms of the CC‐BY 4.0 license [54]. Copyright 2022, The Authors, published by Frontiers. E) Representative physicochemical characteristics of quercetin‐based supramolecular hydrogels. Reproduced under the terms of the CC‐BY 4.0 license [52]. Copyright 2025, The Authors, published by Wiley‐VCH GmbH. F) Multimodal assessment of wet‐adhesive performance of quercetin‐based supramolecular hydrogels. Reproduced under the terms of the CC‐BY 4.0 license [52]. Copyright 2025, The Authors, published by Wiley‐VCH GmbH.

Quercetin, a flavonoid commonly found in teas and onions, functions as a potent antioxidant. Quercetin is biosynthesized through the phenylpropanoid metabolic route and exhibits broad pharmacological activities, including anti‐inflammatory, antioxidant, and immunomodulatory properties [59]. Quercetin has emerged as a promising therapeutic candidate for wound healing, attributed to its potent antioxidant and anti‐inflammatory activities. Recent investigations have further validated its efficacy in promoting tissue repair in rat cutaneous wound models [60]. In addition, quercetin inhibits astrocyte migration and proliferation by regulating ERK1/2 and FAK, which may help reduce astrocyte scarring in vivo [61]. At the molecular level, past studies have reported the role of quercetin in inhibiting MAPK under different types of inflammatory conditions. Quercetin inhibits the secretion of inflammatory cytokines in both adipocytes and macrophages by inhibiting MAPK signaling factors ERK1/2, JNK, and p38 MAPK in obesity‐induced inflammatory responses [41]. Importantly, quercetin directly alters hydrogel gelation kinetics and viscoelastic behavior via host‐guest interactions, as illustrated by its mechanical and rheological effects in supramolecular hydrogels, Figure 4E [52]. Moreover, the wet‐adhesive performance of quercetin‐based hydrogels, which is crucial for wound adherence in moist environments, is demonstrated by their high burst‐pressure sealing capacity and tissue adhesion behavior, Figure 4F [52]. Likewise, a quercetin‐loaded supramolecular hydrogel adhesive has been shown to provide sustained antioxidant and anti‐inflammatory activity while enhancing barrier restoration and collagen remodeling in hemorrhagic cystitis models. Although developed for bladder mucosal injury, its ability to regulate NF‐κB‐mediated inflammation, promote angiogenesis, and support wound‐site matrix repair highlights the broader wound‐healing potential of quercetin‐based systems. Apigenin (C_15_H_10_O_5_) is a natural bioactive compound belonging to the class of flavones. Widely distributed in various fruits, vegetables, and herbs, apigenin exhibits potent antioxidant properties, neutralizing free radicals and ROS to combat oxidative stress and cellular damage [62]. Its anti‐inflammatory effects are attributed to the inhibition of pro‐inflammatory mediators, suggesting potential in managing conditions like arthritis and inflammatory bowel disease [62]. Applying apigenin topically can enhance the skin's permeability barrier by facilitating processes like epidermal differentiation, the production of antimicrobial peptides, and the synthesis and secretion of lipids. Such an approach might offer advantages in both preventing and treating skin conditions [63]. Furthermore, it exerts similar protective effects on wound tissue by enhancing keratinocyte migration and reducing oxidative damage [64, 65].

In non‐flavonoids, Chlorogenic acid (CGA; C16H18O9), delivered in a hyaluronic acid hydrogel, accelerated diabetic wound closure in mice, reduced inflammatory mediators, and enhanced VEGF/TGF‐β expression, angiogenesis, and collagen deposition and remodeling [66]. Caffeic acid (CA; C9H8O4) and gallic acid (GA; C7H6O5) are phenolic constituents identified in plant extracts with reported anti‐inflammatory activity [67, 68]. CGA has reported antioxidant, anti‐inflammatory, antidiabetic, and blood‐pressure‐lowering effects, whereas CA and CGA may differ in their effects on macrophage fatty‐acid uptake [69, 70]. Curcumin (C_21_H_20_O_6_), derived from Curcuma longa, modulates inflammation, promotes angiogenesis, and inhibits fibrosis through regulation of multiple signaling pathways, such as NF‐κB, TGF‐β, and Smad [39, 40]. Curcumin exerts its protective health effects mainly through anti‐inflammatory and antioxidant mechanisms [71]. More recently, a ZnCur nanoparticle‐enhanced multifunctional hydrogel was shown to combine the anti‐inflammatory properties of curcumin with the antibacterial activity of zinc ions, achieving synergistic immunoregulatory effects that accelerated infected diabetic wound healing [72]. Lignans are a class of non‐flavonoid polyphenols formed by the polymerization of two phenylpropanoid units. These compounds attach to estrogen receptors, act as phytoestrogens, and exhibit anti‐cancer, antioxidant, and other clear pharmacological properties, suggesting their potential for medicinal development [73]. The primary characteristics of lignans include antioxidant, anti‐inflammatory, anti‐cancer, neuroprotective, anti‐fibrosis, among others, with pathways like Keap1/Nrf2, NF‐κB, PI3K/Akt, MAPK, TGF‐β/Smad, and AMPK playing a role [74, 75, 76]. Schisandrin B has been reported to attenuate LPS‐induced sepsis through miR‐17‐5p‐mediated downregulation of TLR4 [73, 77].

#### 3.1.2 Terpenoids

Terpenoids are considered to be a very diverse group of natural compounds with a wide range of biological activities, found widely in plants as constituents of essential oils [78, 79]. Also known as isoprenoids, they are the most numerous and structurally diverse class of natural products [80]. The majority of terpenoids possess complex, multicyclic formations, distinct in their functional groups and fundamental carbon frameworks. The fundamental component of their structure is the hydrocarbon isoprene, represented as CH_2_═C(CH_3_)─CH═CH_2_ [81]. Terpenes display a range of pharmacological characteristics, including blood pressure reduction, antimicrobial effects, anti‐inflammatory properties, antioxidant properties, pain relief, and itch‐relieving properties. Several terpenes have been reported to suppress microbial RNA and protein synthesis with antimicrobial properties, reducing the production of tumor necrosis factor (TNF)‐α and interleukin (IL)‐6 in mast cells, curtailing Leukotriene C4 release, and influencing Thromboxane B2 release through anti‐inflammatory and antioxidant actions by blocking UVB‐triggered free radical generation [47]. Their cytoprotective potential also helps the healing process [82]. Within the context of wound healing, they serve as sophisticated modulators, particularly during the critical tissue remodeling phase. Asiaticoside (C_48_H_78_O_19_), extracted from Centella asiatica, is renowned for its properties in preventing keloid and hypertrophic scarring [83]. It may exert its effects by suppressing the expression of fibrotic genes including collagen I, collagen III, TGF‐β1, and interleukins 1β, 6, and 8, while enhancing the expression of SMAD7 and PPAR‐γ [83]. An asiaticoside‐loaded dual‐crosslinked hydrogel has recently been shown to reduce inflammation, promote angiogenesis, accelerate healing in diabetic foot ulcers (reducing the residual wound area to 8.9 ± 2.1% by day 14, compared with 31.5 ± 9.4% in the untreated control) and illustrates how hydrogel systems can markedly enhance the stability and therapeutic performance of terpenoid compounds in chronic wound environments [48]. Consistently, an injectable recombinant collagen/quaternary‐ammonium chitosan hydrogel co‐delivering asiaticoside‐loaded liposomes and a silver‐loaded metal–organic framework (Ag@MOF) enabled sustained AS/Ag^+^ release, enhanced angiogenesis and antibacterial activity, and accelerated healing in a burn‐infected wound model, Figure 5A [49]. Building on this, a co‐assembled asiaticoside and panax notoginseng saponin hydrogel has further demonstrated that PNS can stabilize AS and enhance its bioavailability, leading to synergistic anti‐inflammatory and pro‐angiogenic effects through IL‐6 suppression, VEGF upregulation, and improved collagen remodeling (raising the wound‐healing rate to 88 ± 4.4% by day 14, comparable to the 90.2 ± 3.8% of the positive control) [84]. Similarly, a glycyrrhizic acid‐based supramolecular hydrogel has also been reported, featuring a robust dual‐network architecture that provides injectable, self‐healing, and adhesive properties. Benefiting from the intrinsic anti‐inflammatory and antibacterial activities of this natural saponin, the hydrogel markedly enhanced granulation tissue formation, collagen deposition, and infection control, thereby accelerating both sterile and S. aureus‐infected wound healing [85]. Glycyrrhizic acid has also been used as a co‐assembling partner to form a carrier‐free small‐molecule hydrogel with a cryptotanshinone‐peptide conjugate, showing high skin permeability with synergistic antibacterial and anti‐inflammatory effects in acne models, Figure 5B [86]. Likewise, a tea saponin loaded CMC hydrogel enabled sustained release of this triterpenoid saponin, providing strong antibacterial activity and accelerating acute wound healing. Its tough, adhesive network further contributed to hemostasis and inflammation control, highlighting its potential for treating irregular or highly mobile wounds [87].

**FIGURE 5:**
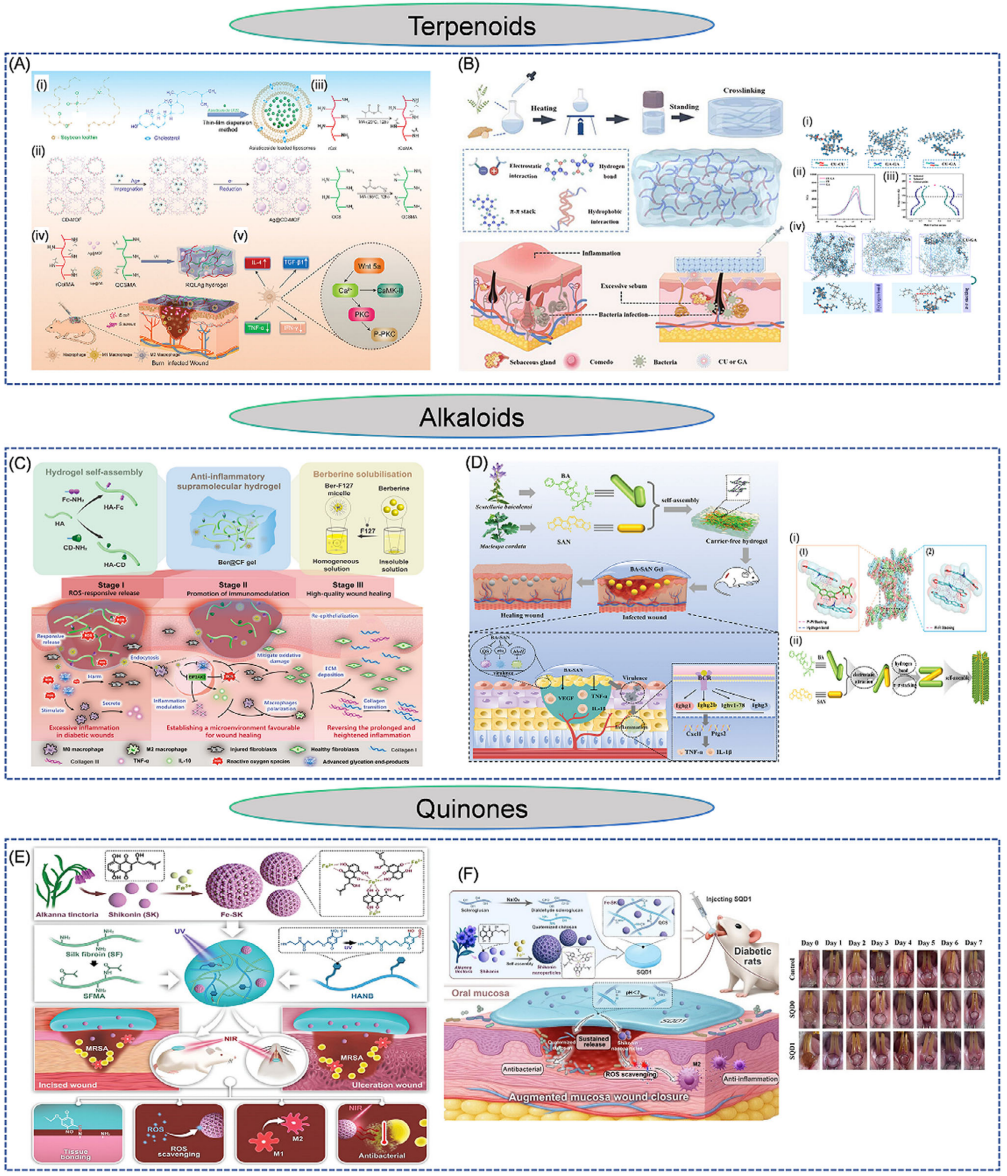
Representative hydrogel delivery strategies for terpenoids, alkaloids, and quinones in wound and skin infection therapy. A) An injectable hydrogel delivering a terpenoid and an antibacterial component for infected wound repair. Reproduced with permission [49]. Copyright 2023, Wiley‐VCH GmbH. B) A small molecule self‐assembled hydrogel formed by two plant derived compounds for topical anti‐inflammatory and antimicrobial skin therapy. Reproduced with permission [86]. Copyright 2025, American Chemical Society. C) A reactive oxygen species responsive supramolecular hydrogel enabling on‐demand release of an alkaloid to improve diabetic wound healing. Reproduced with permission [88]. Copyright 2025, Elsevier. D) A carrier free self‐assembled hydrogel combining two plant bioactives with synergistic antibacterial activity for infected wounds. Reproduced with permission [42]. Copyright 2022, Wiley‐VCH GmbH. E) A light‐activated adhesive hydrogel patch incorporating a quinone for rapid infection control and mucosal wound closure. Reproduced under the terms of the CC‐BY 4.0 license [44]. Copyright 2024, The Authors, published by Wiley‐VCH GmbH. F) An injectable biodegradable polysaccharide hydrogel incorporating a quinone‐based component to reduce bacterial burden and oxidative stress in diabetic oral ulcers. Reproduced with permission [89]. Copyright 2026, Elsevier.

Despite these promising activities, most terpenoids are poorly water‐soluble and rapidly cleared from the wound site, which limits their direct topical application. Hydrogel encapsulation (discussed in detail in Section 4) offers a practical route to improve local retention and control release kinetics for this compound class.

#### 3.1.3 Alkaloids

Alkaloids are nitrogen‐containing plant secondary metabolites characterized by one or more heterocyclic nitrogen atoms in their core structure [90]. Classification of these molecules is determined by the unique heterocyclic ring architectures that characterize their chemical structures. They constitute critical pharmacologically active constituents in Chinese herbal therapeutic systems and manifest significant bioactivity profiles. The alkaloid research domain has witnessed substantial expansion driven by enhanced natural product extraction techniques and continual development of innovative technological approaches and methodological frameworks. Alkaloids derived from traditional plants medicine with bioactive properties, Vincristine (C_46_H_56_O_10_) and 10‐Hydroxycamptothecin (C_20_H_16_N_2_O_5_) [91], have shown significant effectiveness, for instance. anti‐cancer, anti‐inflammatory, immunosuppressive, and so on [92]. Berberine (C_20_H_18_NO_4_^+^), isolated from Coptis chinensis, is particularly effective in treating wounds infected with drug‐resistant bacteria such as MRSA [46]. It reduces bacterial load, modulates inflammation, and enhances granulation tissue formation through mechanisms involving MAPK and AMPK signaling pathways [93]. To address berberine's poor aqueous solubility and improve retention in oxidative diabetic wounds, berberine was formulated as Pluronic F127 micelles and further loaded into an oxidation responsive cyclodextrin‐ferrocene hyaluronic‐acid supramolecular hydrogel to enable ROS‐triggered, on‐demand release, Figure 5C [88]. Matrine (C_15_H_24_N_2_O) from Sophora flavescens similarly exhibits pronounced anti‐inflammatory and antibacterial activities, underscoring alkaloids’ importance in infection control and immune modulation during wound healing [94, 95]. In addition, total alkaloids from Leonurus japonicus have been shown to accelerate wound repair by enhancing cellular proliferation, collagen deposition, and angiogenesis through SRC/MEK/ERK signaling, further supporting the complex regenerative potential of alkaloid compounds in cutaneous healing (in a rat full‐thickness wound model, the healing area reached approximately 50% by day 6, with near‐complete closure by day 15) [50]. Beyond single‐agent delivery, a carrier‐free baicalin‐sanguinarine self‐assembled hydrogel formed via electrostatic attraction, π–π stacking, and hydrogen bonding showed synergistic anti‐MRSA activity and mitigated inflammation driven by virulence factors, accelerating infected wound healing, Figure 5D [42].

However, alkaloids are poorly stable, often undergo N‐oxidation, and are also affected by heat, light, and solvents. Alkaloids are water‐soluble under acidic conditions and fat‐soluble under neutral or alkaline conditions. Alkaloids have a limited oral bioavailability, a short half‐life and a fast clearance rate, which alters their efficacy. To overcome these physicochemical disadvantages, researchers have used continuous delivery (gels), transdermal delivery, or penetration enhancer to improve their properties. Similarly, berberine‐loaded κ‐carrageenan hydrogels incorporating selenium nanocomposites have demonstrated enhanced antibacterial, anti‐inflammatory, and pro‐regenerative effects in MRSA‐infected wounds, further highlighting the therapeutic potential of alkaloid‐based hydrogel systems in complex infectious wound environments [46]. Matrine‐loaded self‐healing hydrogels combined with exosomes have been shown to suppress bacterial growth, rebalance inflammatory macrophage phenotypes, and stimulate neovascularization in diabetic wounds, providing a more stable microenvironment for rapid tissue regeneration (with complete wound closure achieved by day 14, whereas the control groups retained substantially larger open wound areas) [51].

#### 3.1.4 Quinones

Quinones are natural organic substances characterized by the presence of an unsaturated cyclic diketone quinone structure or the ease of conversion to such a structure [96]. Many quinones, characterized by their conjugated cyclic diketone structures, are known for strong redox properties, contributing significantly to their antimicrobial and pro‐regenerative functions [96, 97]. Shikonin (C_16_H_16_O_5_), sourced from Lithospermum erythrorhizon, demonstrates substantial effectiveness in promoting fibroblast and endothelial cell proliferation, antimicrobial activity, and suppression of excessive inflammation, which facilitates rapid and effective wound closure [98]. A wet‐adhesive photocurable patch integrating ferric iron/shikonin nanoparticles within an ECM‐mimicking SFMA/HA‐NB matrix further prolonged local coverage and delivered combined antibacterial, anti‐inflammatory, and ROS‐regulating effects in diabetic infected mucosal wounds, Figure 5E [44]. Emodin (C_15_H_10_O_5_) and Tanshinone IIA (C_19_H_18_O_3_) exhibit comparable effects, notably through their capacity to modulate oxidative stress, inhibit excessive fibroblast activation, and enhance angiogenesis.

However, the very redox activity that makes quinones effective also presents a significant challenge: the potential for off‐target effects and dose‐dependent cytotoxicity. The line between therapeutic ROS generation and damaging oxidative stress to healthy cells can be thin. Therefore, the development of hydrogel‐based delivery systems is particularly critical for quinones, an injectable polysaccharide hydrogel reinforced with shikonin nanoparticles provided antimicrobial activity and efficient ROS scavenging, helping shift diabetic oral ulcers from chronic inflammation toward a proliferative healing state, Figure 5F [89]. Such platforms can precisely control the local concentration and release kinetics, ensuring that their potent bioactivity is harnessed for tissue regeneration while mitigating the risk of toxicity, keeping the local dose within the effective‐but‐safe window. Table 2 summarizes the rational hydrogel design strategies and specific implementation forms for each phytochemical class.

**TABLE 2:** Rational Design Strategies for Incorporating Phytochemical Classes into Hydrogel Matrices.

Phytochemical	Specific bioactivity and therapeutic target in cited study	Physicochemical challenge addressed in study	Rational design strategy and implementation Form	Refs.
1.Polyphenols				
1.1 Flavonoids				
Quercetin	Inflammation regulation: Inhibits NF‐κB pathway to treat hemorrhagic cystitis.	Solubility and gelation interference: Hydrophobicity disrupts hydrogel network formation.	Host–Guest assembly: Supramolecular integration into β‐cyclodextrin cavities to modulate viscoelasticity and ensure sustained release.	[52]
	Antibacterial and angiogenesis: Eliminates S. aureus/E. coli and promotes collagen synthesis.	Stability: Rapid degradation in physiological pH.	Delivery limitation: Low water solubility and rapid in‐vivo metabolism limit bioavailability.	[99]
	Scarless healing: Downregulates TNF‐α/IL‐6; promotes collagen deposition in burn wounds.	Bioavailability: Poor solubility limits topical efficacy.	Hydrogen bonding integration: Incorporated into oxidized alginate/chitosan hydrogel via hydrogen bonding for sustained release.	[100]
Apigenin	Green synthesis: Antioxidant activity for general wound healing.	Extraction efficiency: Traditional extraction uses toxic solvents.	Green synthesis: Ultrasonication‐assisted green‐solvent extraction from whole tomatoes, optimized via response‐surface/ANN modeling, followed by hydrogel formation.	[101]
Baicalin	Synergistic antibacterial: Inhibits MRSA virulence factors; Alleviates inflammation.	Carrier compatibility: Hard to load high doses without precipitation.	Carrier limitation: Conventional carrier excipients introduce safety risks and high translational costs.	[42]
1.2 Non‐Flavonoids				
Curcumin	Flap survival: Promotes neovascularization; Attenuates ischemia/hypoxia oxidative stress.	Cellular uptake: Poor cell membrane penetration and solubility.	Exosome‐biomimetic delivery: Loaded into exosomes (membranous vesicles) within a polysaccharide hydrogel for targeted intracellular delivery.	[102]
	Nerve regeneration: Promotes Schwann cell migration; Modulates neuro‐immune environment.	Delivery control: Needs precise timing to match nerve repair.	Conductive NIR‐responsive network: polyaniline‐grafted QCS hydrogel with NIR‐triggered photothermal release for nerve‐repair‐synchronized therapy.	[103]
Tannic Acid	Vascularization: Translates mechanical cues to cells.	Mechanical signal: Lacks intrinsic mechano‐transduction ability.	Molecular bridge: Links magnetic nanoparticles to the matrix, converting magnetic fields into mechanical forces for cells.	[104]
2. TERPENOIDS				
Asiaticoside	Burn repair: Promotes angiogenesis; Antibacterial (via Ag synergism).	Bioavailability: High molecular weight limits penetration.	Liposomal delivery: Encapsulated in liposomes within a recombinant collagen network to enhance stability and penetration.	[49]
Glycyrrhizic Acid	Acne treatment: Synergistic anti‐inflammatory with Cryptotanshinone.	Skin permeability: Needs to penetrate pilosebaceous units.	Small‐molecule co‐assembly: Forms a carrier‐free, supramolecular hydrogel with high skin permeability.	[86]
3. ALKALOIDS				
Berberine	MDR bacteria eradication: Synergizes photodynamic therapy with chemotherapy to eliminate antibiotic‐resistant bacteria (e.g., S. aureus, P. aeruginosa) and promote infected wound healing.	Drug resistance and solubility: Single‐mode therapy is ineffective against resistant strains; hydrophobic nature limits local bioavailability.	Photo‐responsive nanocomposite: Berberine is encapsulated into porphyrin‐based MOF nanoparticles and embedded in a chitosan hydrogel to achieve light‐triggered release and ROS generation.	[105]
	Diabetic wound: Immunomodulation; Microenvironment reconstruction.	Solubility: Poor aqueous solubility.	Micelle‐in‐hydrogel: Formulated as polymeric micelles loaded into a ROS‐responsive hydrogel.	[88]
Matrine	Angiogenesis and immunomodulation: Promotes vascularization and rebalances inflammatory macrophages in diabetic wounds.	Short half‐life: Rapid clearance from the wound site requires frequent administration.	Delivery limitation: Free exosomes have short in‐vivo half‐life and rapid clearance; matrine benefits from localized sustained delivery via exosome encapsulation.	[51]
Sanguinarine	Biofilm suppression: Disrupts Staphylococcus pseudintermedius biofilms and inhibits EPS synthesis.	Biofilm penetration: Difficult to penetrate mature bacterial biofilms.	Hydrogel‐based delivery: A formulated hydrogel that maintains local concentration to disrupt biofilm integrity and suppress virulence factors.	[45]
4. QUINONES				
Shikonin	Oral mucosa repair: Promotes keratinocyte proliferation.	Cytotoxicity: Narrow therapeutic window (toxic at high doses).	Nanoparticle reinforcement: Engineered as Fe/Shikonin nanoparticles to strictly control concentration and scavenge ROS.	[44]
Emodin	Infected wound healing: Synergistic antibacterial effect against S. aureus and E. coli.	Extreme hydrophobicity: Very poor water solubility hinders hydrogel incorporation.	Eutectogel formation: Solubilized via Schiff base reaction with chitosan oligosaccharides to form a “Eutectogel,” significantly enhancing solubility and bioavailability.	[106]

As outlined in Table 2, each phytochemical class faces distinct physicochemical barriers that prevent direct clinical application: polyphenols suffer from poor aqueous solubility and oxidative instability, terpenoids are rapidly cleared from the wound site, alkaloids undergo N‐oxidation and have short half‐lives, and quinones present narrow therapeutic windows due to dose‐dependent cytotoxicity. These class‐specific limitations define the design requirements that hydrogel carriers must satisfy. Section 4 therefore presents the hydrogel strategies that have been rationally matched to these requirements, progressing from endogenous stimuli‐responsive networks (Section 4.1) to externally triggered release systems (Section 4.1.5) and finally to 3D‐printed architectures for spatially controlled delivery (Section 4.2).

## 4 Rational Design of Phytochemical‐Hydrogel Systems: From Stimuli‐Responsiveness to 3D Printing

### 4.1 Stimuli‐Responsive Systems

Stimuli‐responsive hydrogels can be broadly divided into two mechanistic categories according to the origin of their activating signal. Endogenous (internally triggered) systems respond to pathological cues intrinsic to the chronic wound bed, namely the alkaline pH shift caused by bacterial colonization (Section 4.1.1), the excess reactive oxygen species generated by sustained oxidative stress (Section 4.1.2), the persistent hyperglycemia of diabetic ulcers (Section 4.1.3), and the abnormally elevated protease activity of non‐healing wounds (Section 4.1.4). Because their trigger is the disease state itself, these systems release their payload autonomously and in proportion to wound severity, but afford the clinician little temporal control. Exogenous (externally triggered) systems, by contrast, respond to physical stimuli applied by the operator, such as heat, light, electrical current, or magnetic fields (Section 4.1.5), and therefore provide precise, on‐demand, clinician‐controlled dosing, at the cost of requiring external hardware and patient compliance. Table 3 maps each trigger to the dominant wound pathology it is designed to address. The integration of natural phytochemicals into stimuli‐responsive hydrogel networks offers a novel strategy in wound therapeutics, enabling intelligent drug delivery systems that respond dynamically to the complexities of the wound microenvironment. These smart materials use both endogenous wound signals (Figure 6A) [10], including pH fluctuations (Figure 6B) [107], ROS elevation (Figure 6C) [108], glucose dysregulation (Figure 6D) [109], and protease overexpression (Figure 6E) [110] and externally applied stimuli such as temperature modulation (Figure 6F) [111], light irradiation (Figure 6G) [112], electrical stimulation (Figure 6H) [113], and magnetic (Figure 6I) [114] fields to achieve spatiotemporally controlled release of therapeutic compounds derived from medicinal plants [115, 116, 117]. The principal stimuli, responsive mechanisms, and representative phytochemical implementations discussed in the following subsections are consolidated in Table 3.

**TABLE 3:** Stimuli‐Responsive Hydrogel Mechanisms for Phytochemical Delivery: Triggers, Mechanisms, and Therapeutic Benefits.

Primary stimulus	Responsive mechanism	Implementation and multi‐response note	Therapeutic benefit	Refs.
pH	Schiff base hydrolysis: Dynamic imine bonds (‐C═N‐) cleave in acidic environments.	PVA‐borax/resveratrol‐grafted cellulose nanofibril network: Faster release under acidic conditions (64.2% at pH 5.4 vs 27.6% at pH 7.4, 48 h)	Infection‐targeted: High‐dose release localized to infection sites, minimizing systemic toxicity.	[124]
	Supramolecular disassembly: Peptide self‐assembly forces (electrostatic/H‐bond) weaken at acidic pH.	Peptide‐curcumin co‐assembly: A cationic peptide (Nap‐FFKKK) co‐assembles with Curcumin at pH 7.8 but releases it at pH 5.5 (Single: pH).	Biofilm eradication: Targeting the acidic microenvironment of MRSA biofilms.	[181]
ROS	Boronate ester exchange/oxidation: PBA forms dynamic bonds that break under high ROS or Glucose.	G‐quadruplex supramolecular hydrogel: A guanosine‐PBA network loaded with Quercetin disintegrates upon ROS oxidation or Glucose competition (Dual: ROS+Glucose).	Diabetic precision: Synchronizes antioxidant release with both oxidative stress and hyperglycemic peaks to rescue stalled wounds.	[142]
	Boronate ester oxidation: PBA oxidizes to phenol, disrupting crosslinks.	HA‐Tannic Acid Hydrogel: High ROS levels collapse the network, releasing Tannic Acid to scavenge free radicals (Multi: ROS+pH+Glucose).	Antioxidant cascade: The material consumes harmful ROS to trigger the release of repair agents.	[182]
	Boronate‐ester cleavage	PBA‐modified quaternized‐chitosan/PVA hydrogel releases procyanidin‐Ce/Cu bimetallic nanoparticles under acidic/high‐ROS conditions	Synergistic sterilization: Synchronizes release with the peak of bacterial oxidative burst.	[135]
Glucose	Competitive binding: Glucose displaces polymer chains from Boronate Esters.	Chitosan‐PBA Matrix: High glucose concentration disrupts crosslinks, releasing Salvianolic Acid B (Dual: Glucose+ROS).	Diabetic precision; couples with angiogenesis.	[138]
Exogenous and Bio‐Specific Triggers	Photothermal effect: Nanoparticles convert NIR light to heat, softening the matrix.	Fe/Shikonin Nanoparticles: NIR light triggers heat generation and Shikonin release from a silk fibroin patch (Dual: Light+pH).	Remote control: Clinician‐controlled “On‐Demand” sterilization and thermal ablation of bacteria.	[44]
	Triboelectric effect: Motion generates voltage, disrupting electrostatic bonds.	Body motion generates current, causing PPy reduction/contraction and curcumin.	Rehabilitation: Couples physical rehabilitation movements may complement physical rehabilitation.	[161]
	Temperature + pH + NIR.	Genipin‐crosslinked CMC hydrogel with Cur‐F127 micelles and PDA nanoparticles.	Acid/heat/NIR‐triggered release.	[183]

**FIGURE 6:**
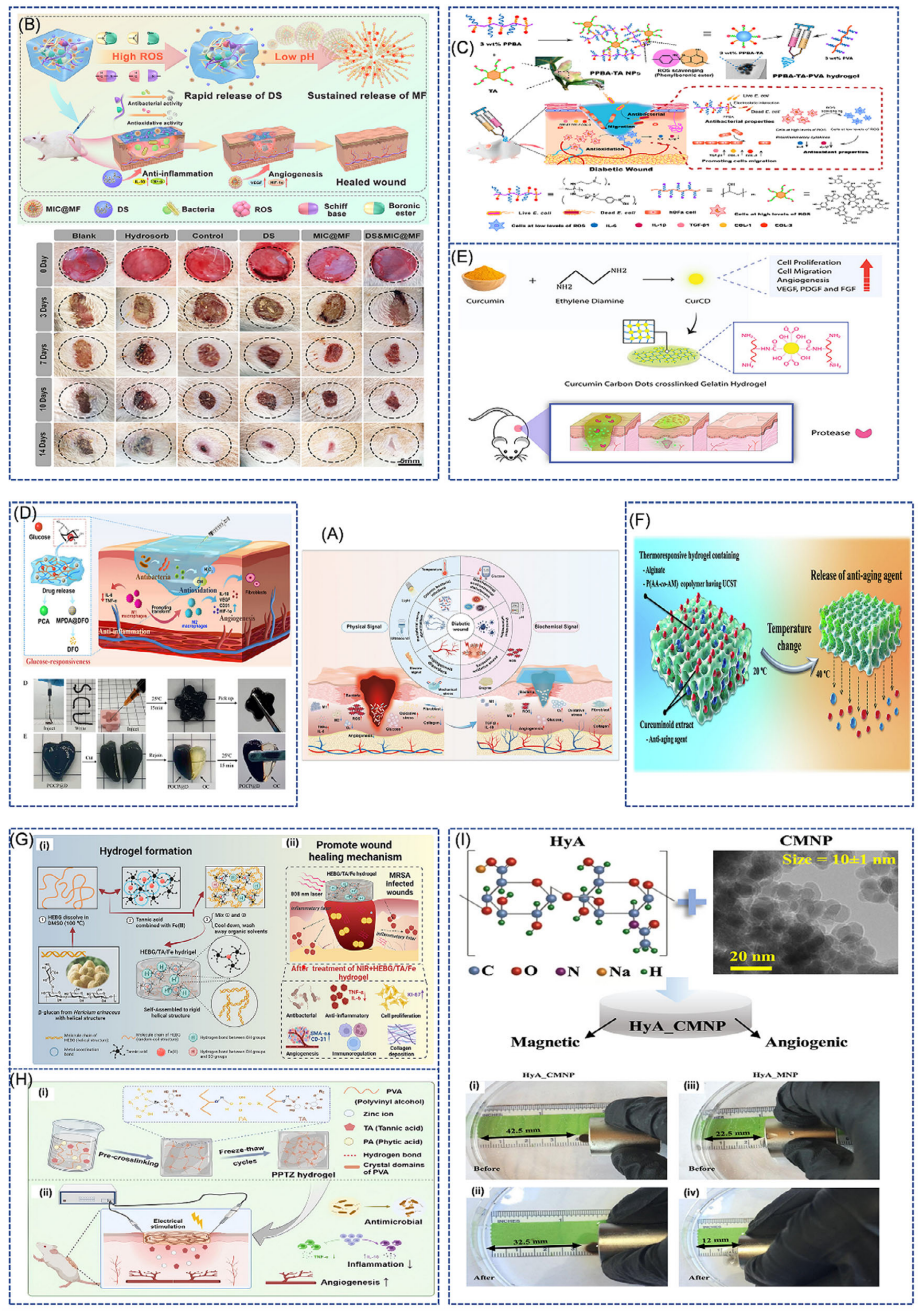
Representative stimuli‐responsive phytochemical hydrogel systems for wound therapy. A) Conceptual overview of smart hydrogels that integrate endogenous wound cues and exogenous triggers to enable on‐demand, spatiotemporally controlled phytochemical delivery. Reproduced with permission [10]. Copyright 2025, Royal Society of Chemistry. B) pH/ROS associated microenvironment responsive release behavior and therapeutic outcomes in an infected diabetic wound model. Reproduced with permission [107]. Copyright 2022, Elsevier. C) ROS scavenging phenylboronic acid/tannic acid, based nanoparticle hydrogel dressing enabling antioxidation/antibacterial functions and wound repair. Reproduced with permission [108]. Copyright 2022, American Chemical Society. D) Glucose‐responsive, injectable/self‐healing hydrogel system. Reproduced with permission [109]. Copyright 2025, American Chemical Society. E) Protease‐responsive gelatin hydrogel crosslinked by curcumin derived carbon dots to enhance stability/solubility and achieve sustained local bioactivity. Reproduced with permission [110]. Copyright 2022, Elsevier. F) Semi‐interpenetrating polymer network hydrogel for controllable release of curcuminoid extracts. Reproduced with permission [111]. Copyright 2025, Elsevier. G) Light‐activated hydrogel strategy with mechanism‐oriented infection control and healing promotion. Reproduced with permission [112]. Copyright 2025, Elsevier. H) Conductive hydrogel dressing combined with electrical stimulation to facilitate cell migration and accelerate diabetic wound closure. Reproduced with permission [113]. Copyright 2024, Tsinghua University Press. I) Magnetic hyaluronic acid hydrogel incorporating curcumin‐containing magnetic nanoparticles to couple angiogenic activity with magnetic guidance/stimulation. Reproduced with permission [114]. Copyright 2022, American Chemical Society.

#### 4.1.1 PH‐Responsive Release

Hydrogels responsive to pH are engineered to react to environmental pH fluctuations, resulting in changes to their structure, swelling behavior, or drug release profiles [118]. pH‐responsive hydrogel systems exploit the characteristic pH gradients in wound environments: normal skin maintains a pH of 4–6, while chronic wounds often exhibit alkaline conditions (pH 7.3–8.9) due to bacterial colonization [119, 120]. When an acute wound occurs, the pH of the tissue and interstitial fluid in the body is about 7.4, which changes the acidic milieu of normal skin and reverts to acidic as wound healing [121]. Consequently, pH levels are deemed a dependable indicator of wound status, with pH fluctuations reflecting the likelihood of wound healing or deterioration. Notably, pH‐responsive wound dressings offer unique advantages in wound treatment due to their superior biochemical and mechanical properties, along with their ability to adjust their shape, size, and drug release behavior. These pH‐responsive dressings can monitor wound status based on pH levels, facilitate controlled healing, reduce the risk of infections, and expedite the healing process [121, 122, 123]. Mechanistically, pH‐responsive antibacterial hydrogel dressings typically couple pH‐dependent network swelling/charge switching with pH‐labile interactions to coordinate antimicrobial function and microenvironment regulation, Figure 7B [121].

**FIGURE 7:**
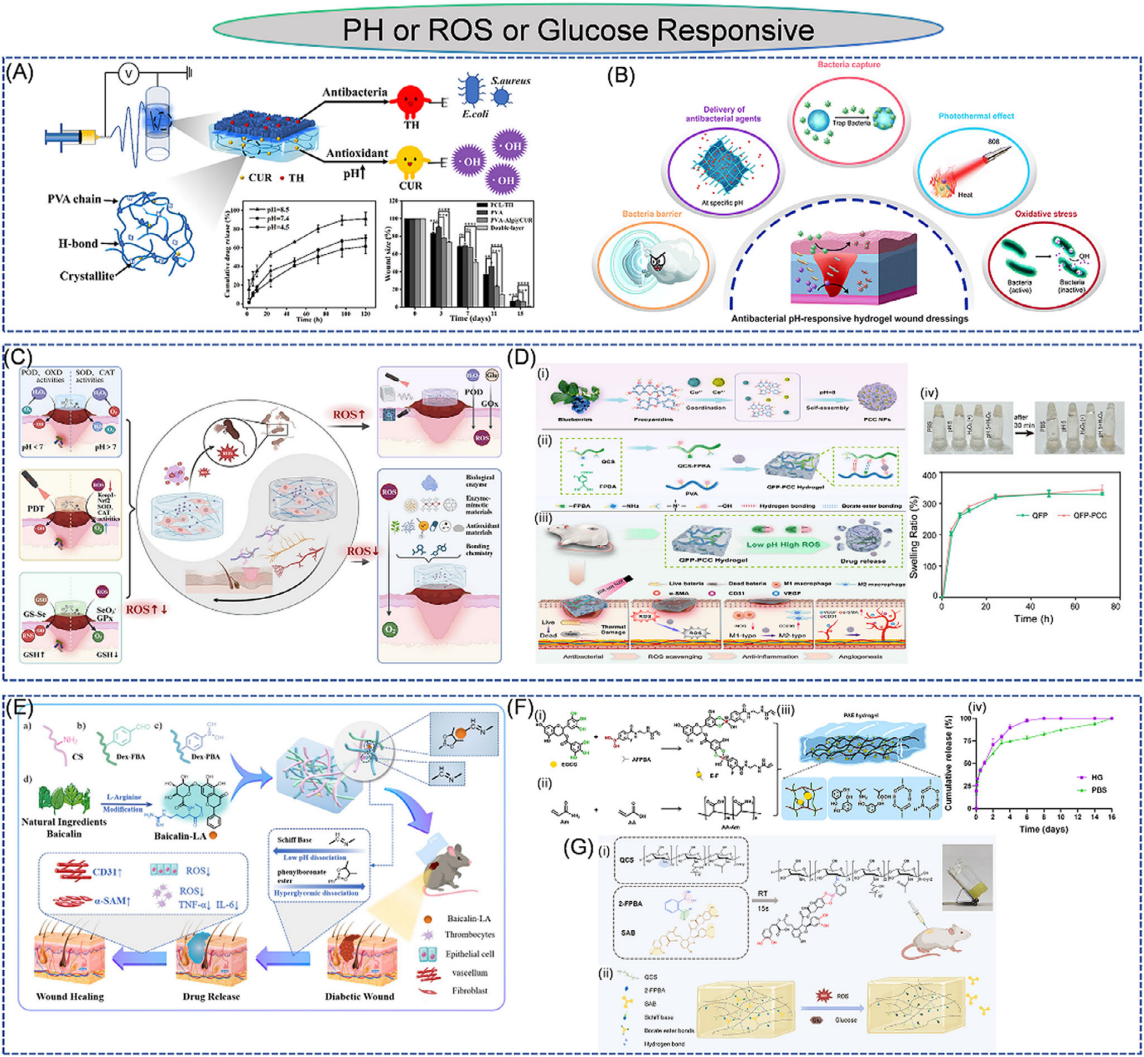
pH, ROS‐, and glucose‐responsive natural phytochemicals hydrogel platforms for chronic wound management. A) A bilayer dressing enabling sequential antibacterial release and pH‐gated antioxidant delivery. Reproduced with permission [126]. Copyright 2024, Elsevier. B) Common antibacterial mechanisms of pH‐responsive hydrogel wound dressings. Reproduced with permission [121]. Copyright 2023, Royal Society of Chemistry. C) Design concepts for hydrogels that regulate ROS in diabetic wounds, including bidirectional redox tuning. Reproduced with permission [134]. Copyright 2025, Wiley‐VCH GmbH. D) A dual pH/ROS responsive hydrogel enabling on‐demand release and multifunctional diabetic wound repair. Reproduced with permission [135]. Copyright 2025, Elsevier. E) A dynamic polysaccharide hydrogel incorporating a baicalin derivative to mitigate oxidative stress and inflammation. Reproduced with permission [136]. Copyright 2024, Elsevier. F) A polyphenol boronate ester hydrogel enabling sustained EGCG delivery with broad pro‐healing effects. Reproduced with permission [137]. Copyright 2024, Elsevier. G) A dual glucose/ROS responsive injectable hydrogel enabling controlled release of salvianolic acid B and coordinated immunomodulation and angiogenesis. Reproduced with permission [138]. Copyright 2025, Elsevier.

Recent advances use this pH heterogeneity through sophisticated chemical mechanisms to achieve targeted phytochemical delivery. An innovative pH‐responsive hydrogel (RPC/PB) has been developed, which combines poly(vinyl alcohol)‐borax (PB) with naturally occurring antibiotic resveratrol‐grafted cellulose nanofibrils (RPC) for the management of bacterial‐infected wounds [124]. Notably, the RPC/PB hydrogel, once exposed to wound‐specific pH, exhibits pH‐dependent drug release: the total release of resveratrol at pH 5.4 is 2.33 times higher than that at pH 7.4, enabling effective adaptation to the acidic wound environment [124]. An injectable, self‐healing QCS/PF127‐CHO micelle‐hydrogel with antibacterial, hemostatic, adhesive, and pH‐responsive release properties matches the mechanical properties of skin. When loaded with curcumin, it significantly accelerates full‐thickness wound healing in vivo, indicating strong potential for joint wound care [125]. A bilayer electrospun‐hydrogel dressing separated a fast‐releasing antibiotic layer from a pH‐gated curcumin reservoir, enabling early infection control and pH‐adaptive antioxidant support in infected diabetic wounds, Figure 7A [126]. A key breakthrough lies in the use of Schiff base chemistry, which relies on dynamic covalent bonds between amino and aldehyde groups [127, 128]. Ninan et al. [129]. developed a tannic acid‐carboxylated agarose composite hydrogel that exhibits negligible drug release at physiological pH but sustained delivery at acidic wound pH (5.5–6.8). This system achieved 87.9% drug release within 48 h, following Baker–Lonsdale kinetics (R^2^ = 0.989), with carboxylated agarose crosslinked via zinc coordination providing pH‐dependent swelling behavior. This pH sensitivity directly correlates with wound healing outcomes: tannic acid enhances cell migration and suppresses nitric oxide (NO) production in human macrophages. Curcumin delivery via pH‐responsive matrices has emerged as particularly promising [130, 131]. For example, photocrosslinked pectin/gelatin (PeMA/GelMA) hydrogels exhibit 4‐fold faster curcumin release at pH 7.4 than at pH 5.0, making them suitable for targeting infected alkaline wounds. These hydrogels exhibit a compressive modulus of approximately 22 kPa, closely matching native tissue, while maintaining an oxygen permeability of 7.44 mg/mL to support wound tissue respiration [132]. Polyphenolic systems utilizing tannic acid demonstrate exceptional pH responsiveness through carboxylic acid ionization mechanisms. Poly (tannic acid) hydrogels achieve maximum swelling of 1669% at pH 9, with complete degradation within 9 days while maintaining antioxidant capacity throughout the degradation process [133]. The phenolic hydroxyl groups provide multiple benefits: pH‐responsive swelling, antimicrobial activity, and ROS scavenging‐creating multifunctional therapeutic platforms. Additionally, quercetin‐loaded ZIF‐8 nanoparticles embedded in GelMA hydrogels have shown controlled, pH‐regulated quercetin release together with potent anti‐inflammatory, antibacterial, pro‐angiogenic, and pro‐collagen effects, thereby promoting efficient cutaneous wound healing [99].

#### 4.1.2 ROS‐Responsive Release

ROS‐responsive hydrogels employ the profound oxidative gradient distinguishing healthy tissue from the chronic wound microenvironment. Low, tightly regulated ROS levels participate in normal wound signaling, whereas sustained ROS excess contributes to cellular damage and impaired healing in chronic wounds [139]. Elevated ROS can trigger hydrogel degradation or payload release through oxidant‐labile motifs such as thioketals and boronic acids or esters [140]. This mechanism facilitates spatiotemporal therapeutic delivery while concurrently scavenging excess ROS to restore redox equilibrium. Beyond unidirectional scavenging, emerging hydrogel platforms are being designed to bidirectionally tune ROS, lowering pathological oxidative stress while preserving the oxidative set‐points needed for antimicrobial action, Figure 7C [134]. A dual pH/ROS responsive hydrogel showed accelerated degradation and on‐demand payload release under acidic and oxidative conditions, integrating antibacterial, antioxidant, immunomodulatory, and pro‐angiogenic effects for diabetic wound repair, Figure 7D [135].

To address the hypoxic and oxidative duality of diabetic wounds, recent high‐impact strategies have moved beyond simple release to engineer synergistic catalytic cascades. Green tea derivatives like EGCG have been utilized to construct sophisticated oxygen‐supplying systems. Under high oxidative stress, the EGCG shell preferentially degrades, scavenging superficial ROS to protect the microenvironment. This degradation subsequently exposes the CaO_2_ core, triggering the controlled generation of molecular oxygen. This cascade not only neutralizes the inflammatory oxidative storm but also alleviates tissue hypoxia, driving high‐quality angiogenesis and M2 macrophage polarization [43]. Older generations of EGCG‐based hydrogels also exploited the phenolic hydroxyl groups as dynamic crosslinkers, creating networks that inherently degrade under oxidative stress to inhibit MMPs [141].

Addressing the solubility barriers of hydrophobic phytochemicals, supramolecular assembly offers a route to high‐efficiency loading without reliance on synthetic surfactants. Quercetin has been successfully integrated into dual‐responsive matrices utilizing guanosine‐PBA networks [142]. These components self‐assemble into G‐quadruplex structures crosslinked by dynamic boronate esters. Upon exposure to pathological ROS or hyperglycemic conditions, the boronate ester bonds dissociate, dismantling the supramolecular scaffold. This dual‐gated mechanism ensures that quercetin is released specifically in response to the diabetic ulcer microenvironment, where it acts synergistically with the matrix to remodel the ECM and accelerate re‐epithelialization [142]. Similarly, Li et al. [143]. developed a double‐network hydrogel that self‐regulated Cu^2^^+^ release at acidic inflammatory pH for antibacterial action and ROS‐responsive luteolin release for sustained antioxidant support, achieving stage‐adaptive chronic wound therapy.

The convergence of nanotechnology with Traditional Chinese Medicine enables sequential modulation of infected wound environments. Baicalein, a flavonoid from Scutellaria baicalensis, has been incorporated into hydrogels via carbon dot technology [144]. These herbal‐derived carbon dots exhibit intrinsic ROS‐responsiveness. The system is engineered to provide a tiered release profile: rapid liberation of antimicrobial agents to disrupt biofilm integrity, followed by the sustained release of baicalein to mitigate residual oxidative damage and promote tissue regeneration. In infected wound models, this nano‐engineered platform demonstrated superior efficacy, reducing wound area to approximately 2% by day 8 through this synchronized sterilize‐and‐repair strategy [144].

#### 4.1.3 Glucose‐Responsive Release

Diabetic chronic wounds are characterized by a hyperglycemic microenvironment (>200 mg/dL) accompanied by elevated oxidative stress and chronic inflammation. Glucose‐responsive hydrogels utilize this pathological glucose level as an endogenous trigger to release therapeutic agents on demand [145]. While early systems focused on insulin delivery, recent strategies increasingly incorporate small‐molecule phytochemicals (e.g., curcumin, gallic acid, epigallocatechin gallate) due to their potent antioxidant and anti‐inflammatory properties. These platforms primarily rely on two mechanisms: PBA dynamic bonding and glucose oxidase (GOx) enzymatic cascades.

Phenylboronic acid‐functionalized hydrogels operate through a reversible chemical equilibrium. PBA groups form cyclic boronate esters with cis‐diol‐containing molecules (such as glucose). In glucose‐containing media, reversible borate ester formation can induce glucose‐dependent network swelling and controlled drug release [145]. In the cited microgel‐composite system, cumulative metformin release exceeded 80% and was sustained for more than 2 days [145]. A dynamically crosslinked polysaccharide hydrogel incorporated a baicalin derivative to attenuate oxidative stress and inflammation, supporting diabetic wound healing through local microenvironment remodeling, Figure 7E [136]. Crucially, many polyphenolic phytochemicals, such as gallic acid and EGCG, naturally contain catechol or pyrogallol groups (cis‐diols). This allows them to serve a dual role: acting as structural crosslinkers to stabilize the hydrogel under normal glucose levels, and acting as released therapeutic antioxidants under hyperglycemic conditions. A polyphenol‐boronate ester hydrogel enabled sustained epigallocatechin gallate release and delivered broad pro‐healing functions in diabetic wounds, including antibacterial and anti‐inflammatory effects, Figure 7F [137]. More broadly, multifunctional antioxidant hydrogels have been developed to reduce excessive ROS in diabetic wounds [146, 147].

The integration of GOx into hydrogels creates a bio‐responsive microenvironment. GOx catalyzes the oxidation of glucose into gluconic acid and hydrogen peroxide (H_2_O_2_). This reaction lowers the local pH, which can trigger pH‐sensitive linkers to release hydrophobic phytochemicals like curcumin. However, the byproduct H_2_O_2_ can exacerbate oxidative stress. To address this, advanced “cascade” systems co‐encapsulate catalase or manganese dioxide (MnO_2_) nanozymes alongside phytochemicals. In these systems, the release of curcumin acts synergistically with the enzymatic consumption of glucose. Studies have shown that such platforms not only reduce local glucose levels but also modulate the immune microenvironment, promoting the polarization of macrophages from the pro‐inflammatory M1 phenotype to the regenerative M2 phenotype [148]. In summary, glucose‐responsive hydrogels loaded with small‐molecule phytochemicals provide a closed‐loop therapeutic strategy. An injectable hydrogel with dual responsiveness to glucose and ROS enabled controlled release of salvianolic acid B in the diabetic wound microenvironment, while coordinating antibacterial, antioxidant, immunomodulatory, and pro‐angiogenic activities, Figure 7G [138]. They simultaneously regulate blood glucose locally and mitigate the oxidative damage inherent to diabetic ulcers.

#### 4.1.4 Enzyme‐Responsive Networks

Enzyme‐responsive hydrogels exploit the protease‐rich microenvironment of chronic wounds, in which MMP and serine‐protease activities remain abnormally elevated relative to acute or healing wounds [14, 149]. This biochemical hallmark provides an ideal endogenous trigger for the spatiotemporal release of hydrophobic phytochemicals, ensuring that potent bioactive agents are liberated strictly at sites of active inflammation, thereby maximizing therapeutic indices while minimizing off‐target effects. The primary strategy involves incorporating enzyme‐labile peptide crosslinkers as molecular gates. For example, the biscysteine sequences CVPGG↓LAGAC and CGPGG↓LAGGC have been incorporated into multi‐arm PEG hydrogels to enable MMP‐9‐triggered degradation and drug release [150]. Related MMP‐cleavable release strategies have also been explored in diabetic wound models [10]. These peptide crosslinkers have been widely incorporated into polymer networks, where the hydrogel remains stable under physiological conditions but undergoes progressive degradation upon exposure to pathological concentrations of wound‐associated MMPs, thereby triggering the on‐demand release of therapeutic payloads specifically at the inflammatory site [151]. For example, researchers have successfully integrated such MMP‐sensitive sequences into oxidized hyaluronic acid and collagen hybrid networks to achieve inflammation‐activated drug delivery in diabetic wound models [152]. In these systems, the hydrogel remains stable under physiological conditions but undergoes rapid degradation upon exposure to pathological concentrations of MMPs, thereby triggering the on‐demand release of therapeutic payloads specifically at the inflammatory site [153]. An inflammation‐activated, protease‐responsive injectable hydrogel enabled sustained local delivery of cinnamaldehyde to protect endothelial function and accelerate diabetic wound repair, Figure 8A [154]. A notable application of this logic involves self‐assembling peptide hydrogels (e.g., MAX8) designed to solubilize and deliver Curcumin, a hydrophobic polyphenol with potent anti‐inflammatory properties. In diabetic wound models, these protease‐sensitive matrices protected the curcumin payload and achieved significant wound closure by synchronizing its release with the local proteolytic activity [155].

**FIGURE 8:**
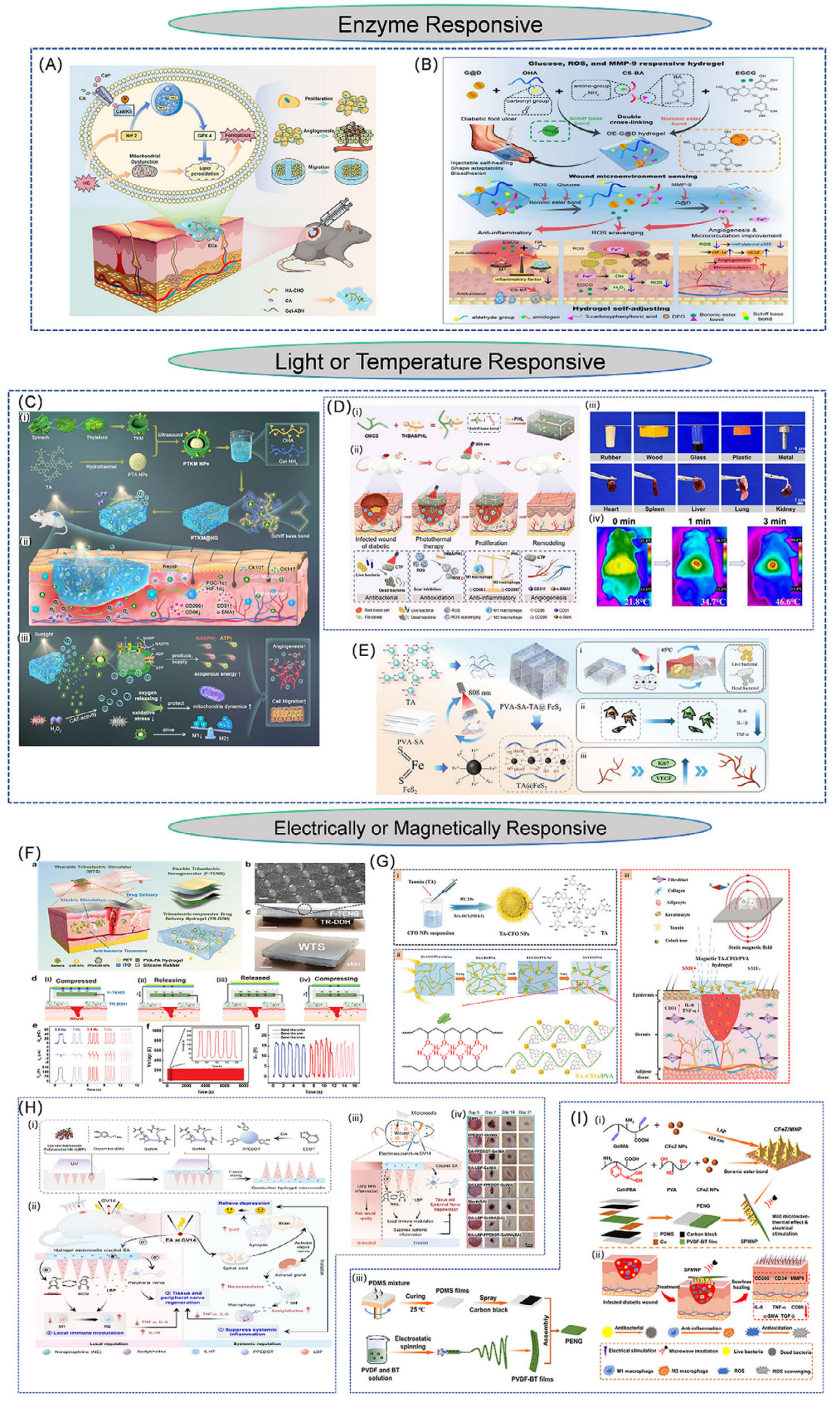
Representative enzyme‐responsive, light/thermal‐triggered, and electrical/magnetic‐assisted phytochemical hydrogel platforms for chronic wound repair. A) Protease‐responsive injectable hydrogel for inflammation‐cued phytochemical delivery. Reproduced with permission [154]. Copyright 2025, Elsevier. B) Microenvironment sensing hydrogel with staged polyphenol and pro‐angiogenic release for diabetic foot ulcers. Reproduced with permission [157]. Copyright 2025, Elsevier. C) Visible light activated hydrogel providing oxygen/bioenergetic support. Reproduced with permission [158]. Copyright 2024, Elsevier. D) NIR‐activated photothermal hydrogel for infected diabetic wounds. Reproduced with permission [159]. Copyright 2025, Ivyspring International Publisher. E) Polyphenol based nanocomposite hydrogel enabling NIR photothermal chemical synergistic therapy. Reproduced with permission [160]. Copyright 2026, Elsevier. F) Wearable self‐powered triboelectric stimulator with a conductive hydrogel electrode for electrical therapy and on‐demand release. Reproduced with permission [161]. Copyright 2024, Wiley‐VCH GmbH. G) Magnetic nanocomposite hydrogel translating static magnetic fields into mechanoactive cues. Reproduced with permission [104]. Copyright 2022, Royal Society of Chemistry. H) Conductive hydrogel microneedles combined with electroacupuncture for coordinated immunomodulation in diabetic wounds. Reproduced with permission [162]. Copyright 2025, Elsevier. I) Self‐powered microneedle patch integrating motion‐derived electrical stimulation and microwave‐assisted mild thermal therapy. Reproduced with permission [163]. Copyright 2025, American Chemical Society.

Phytochemical‐mediated enzyme inhibition networks beyond serving as passive cargo, certain phytochemicals can actively participate in the hydrogel network assembly, exerting a dual function of structural reinforcement and enzymatic inhibition. EGCG acts as a potent natural inhibitor of collagenase, exhibiting 88% inhibition of collagenase activity and conferring 95% resistance of collagen to collagenolytic hydrolysis at micromolar concentrations (10–160 µM) [156]. Recent advances have utilized EGCG as a functional crosslinker to stabilize polysaccharide networks. A microenvironment‐sensing hydrogel integrating hyperglycemia, oxidative stress, and protease‐responsiveness enabled staged release of epigallocatechin gallate followed by a pro‐angiogenic cue, improving microcirculation in diabetic foot ulcers, Figure 8B [157]. For example, EGCG‐crosslinked carboxymethyl chitosan hydrogels create a “shielding effect” that resists enzymatic degradation while providing robust antioxidant capacity [141]. Upon gradual degradation by wound proteases, the released EGCG inhibits collagenase activity [156], while suppressing PI3K/Akt‐mediated ECM breakdown [155], collectively promoting ordered epidermal regeneration.

#### 4.1.5 Externally Triggered Systems

##### 4.1.5.1 Thermo‐ and Light‐Triggered Release

The integration of thermo‐responsive and light‐activated systems enables a move toward non‐invasive, external control in drug delivery for wound care, moving beyond traditional, static dressings [164]. Thermo‐responsive hydrogels typically use the inherent lower or upper critical solution temperature phase transitions that occur near physiological temperatures, enabling precursor solutions to undergo in situ gelation upon administration to the wound bed [164, 165]. This thermal transition is pivotal for controlling initial drug release, ensuring the sustained delivery of encapsulated therapeutics by physically altering the hydrogel's volumetric and network density [164, 165]. Moreover, temperature modulation can soften the hydrogel by breaking dynamic bonds, which increases the material's flexibility, facilitating better adaptation to mobile or irregular wound surfaces and enhancing non‐painful removal [165].

Light responsiveness, particularly utilizing the deep tissue penetration of Near‐Infrared (NIR) light (780–1700 nm), is crucial for enabling PTT as an antibiotic‐free strategy to combat drug‐resistant infections [166, 167]. PTT agents, such as Tannic Acid/Iron chelates (TA/Fe^3+^) or MXene nanosheets modified with polyphenols, efficiently convert NIR light into localized heat [168, 169]. A self‐assembled tannic acid/iron sulfide nanocomposite hydrogel combined polyphenol‐mediated chemical antibacterial action with near‐infrared photothermal ablation to accelerate closure of infected wounds, Figure 8E [160]. A naturally derived hydrogel assembled from polysaccharides and traditional small molecules provided adhesive coverage and, under near‐infrared irradiation, delivered photothermal sterilization together with anti‐inflammatory and pro‐repair effects in infected diabetic wounds, Figure 8D [159]. This localized hyperthermia denatures bacterial proteins, providing potent, remote‐controlled antimicrobial efficacy [169]. For instance, exposure to NIR rapidly raised the temperature of Agarose‐based hydrogels (ATB) containing TA/Fe^3+^ to 53.4 °C in 10 min, successfully eliminating bacteria without causing thermal damage to surrounding healthy tissue [169]. NIR exposure can function as a remote “switch” to trigger the on‐demand release of natural phytochemicals; this mechanism was shown to promote the spatiotemporal release of the alkaloid Berberine Hydrochloride from the ATB hydrogel, leading to a synergistic bactericidal effect that accelerates healing in infected wounds [169]. Similarly, tannic‐acid‐modified MXene hydrogels achieved inhibition rates of 97.7 ± 2.2% against *E. coli*, 96.0 ± 1.7% against S. aureus, and 96.6 ± 1.1% against MRSA under NIR irradiation [168]. In addition, a photo‐modulated hydrogel based on oxidized dextran and ε‐poly‐lysine was shown to deliver nitric oxide under NIR irradiation, combining photothermal ablation with NO‐mediated biofilm dissipation to accelerate MRSA‐infected diabetic wound healing in vivo [170]. Along similar lines, a multifunctional hydrogel incorporating oxygen vacancy‐rich WOx nanowires achieved simultaneous photocatalytic O_2_ production, photothermal antibacterial activity, and glucose consumption under NIR irradiation, addressing the triple challenge of hypoxia, infection, and hyperglycemia in diabetic wounds [171].

Beyond the photothermal mechanism, visible light offers non‐thermal energy input for bioenergetic correction, especially relevant in metabolically compromised chronic wounds like diabetic ulcers. Systems integrating plant‐derived photosynthetic Thylakoid Membranes coated with polyphenolic nanoparticles (PTKM NPs) utilize mild red LED light to continuously generate oxygen and vital energy metabolites (ATP and NADPH) through an artificial photosynthetic effect [158]. This unique light‐driven strategy directly addresses local hypoxia, alleviates oxidative stress via inherent polyphenolic antioxidant activity, and enhances cellular anabolism by correcting mitochondrial dysfunction, culminating in accelerated tissue regeneration and M2 macrophage polarization without causing thermal damage [158]. A visible‐light‐driven hydrogel incorporating plant thylakoid membranes enabled sustained oxygen and bioenergetic support under illumination, helping correct hypoxia‐associated dysfunction in diabetic wounds, Figure 8C [158]. These advanced light and thermal systems collectively demonstrate significant potential by dynamically coupling external control with the pleiotropic benefits of natural phytochemicals, offering sophisticated solutions for complex wound microenvironments.

In short, light and thermal triggers add a layer of clinician‐controlled, on‐demand activation to phytochemical delivery and it's something that purely endogenous stimuli can't provide. The practical trade‐off is the need for external hardware and patient compliance, which must be weighed in clinical design.

##### 4.1.5.2 Electrically and Magnetically Triggered Release

Directly integrating electrical or magnetic responsiveness into hydrogel matrices enables sophisticated control over therapeutic delivery and cellular microenvironment remodeling, fundamentally enhancing healing outcomes [172, 173]. Electroconductive hydrogels (EHCs), utilizing electroactive nanomaterials like polypyrrole (PPy) or PPy‐based composites within natural polymer scaffolds (e.g., chitosan or other polysaccharides), establish biomimetic conductive pathways [103, 172, 174]. This design ensures continuous electrical current conduction, addressing compromised cellular signaling in deep or irregular wounds, a key step for enhancing cell migration and accelerating angiogenesis through electrical stimulation therapy [174]. In a recent study, a skin‐inspired conductive PMQG hydrogel composed of acrylic acid, quaternary ammonium chitosan, and MXene nanosheets demonstrated that coupling with endogenous bioelectric fields could simultaneously achieve broad‐spectrum antibacterial activity and promote angiogenesis in diabetic wounds [175]. Beyond direct electrical stimulation, this inherent conductivity supports the emergence of complex wearable bioelectronic systems. A wearable, self‐powered triboelectric stimulator integrated with a conductive hydrogel electrode enabled microcurrent therapy and facilitated electrically triggered phytochemical release, accelerating healing of bacterially infected wounds, Figure 8F [161]. For instance, conductive hydrogels internally embedded with PPy‐PDA‐MnO_2_ nanoparticles function as epidermal electrodes for real‐time electromyography monitoring of muscle function, facilitating monitoring‐guided, accurate wound rehabilitation critical for DFUs [176, 177]. Polyphenol‐mediated conductive hydrogel microneedles combined with electroacupuncture provided coordinated local and systemic immunomodulation, improving diabetic wound repair while alleviating depressive‐like behaviors, Figure 8H [162]. The therapeutic efficacy of these conductive platforms is synergistically enhanced by integrating natural phytochemicals. For instance, the natural alkaloid Berberine (Ber), loaded into PPy‐modified quaternary ammonium chitosan (QAC) hydrogels, exhibits superior antibacterial performance through the combined mechanism of QAC's intrinsic antimicrobial properties and the potent electrothermal or photothermal effects generated by PPy under external stimulation [103, 178]. Analogously, the plant‐derived polyphenolic compound Resveratrol (Res) imparts antioxidative and proangiogenic functions to conductive hydrogels, addressing pervasive oxidative stress and vascular impairment concurrently with enhanced electrical signaling [176]. A further pivotal example involves incorporating the anti‐inflammatory and neurotrophic phytochemical Curcumin (Cur) into photothermal (PTT) conductive hydrogels [103]. Curcumin's release, triggered on‐demand by NIR irradiation [179], promotes Schwann cell migration and proliferation, thus accelerating peripheral nerve and tissue repair [180].

Conversely, magneto‐responsive hydrogels (MHGs), which integrate magnetic nanoparticles (MNPs) such as Cobalt Ferrite (CFO), provide a non‐invasive method for deep tissue modulation [173]. The external magnetic field serves as a potent trigger, facilitating controlled functions such as pulsed drug release via MNP oscillation, or localized cancer therapy through magnetic hyperthermia [173]. An innovative approach harnesses MNPs for magneto‐mechanical stimulation in chronic wound repair, avoiding the potential thermal or electrical damage linked to other remote field methods [104]. This mechanical modulation strategically relies on the incorporation of natural components acting as efficient stress transducers [104]. Specifically, the natural polyphenol Tannin (TA) acts as a molecular bridge, linking magneto‐deformable CFO nanoparticles to the polyvinyl alcohol (PVA) matrix [104]. A magnetic nanocomposite hydrogel used tannic acid as a molecular bridge between cobalt ferrite nanoparticles and a polymer matrix, translating a static magnetic field into mechanoactive cues that promoted early vascularization and tissue regeneration, Figure 8G [104]. Under a static magnetic field, this TA bridge efficiently transmits the nanoscale deformation originating from the CFO NPs to the hydrogel surface, generating critical, localized mechanical signals perceptible by cells. This precise biophysical modulation substantially accelerates wound healing, significantly promoting early vascularization, and enhancing cell adhesion and proliferation [104]. Furthermore, the TA component inherently offers crucial biochemical cues, exhibiting antibacterial and anti‐inflammatory properties, complementing the regenerative effects provided by Co^2+^ ions released from the magnetic nanoparticles. This synergy results in potent dual therapeutic efficacy from a single magneto‐responsive platform [104].

A self‐powered microneedle patch integrating a piezoelectric nanogenerator achieved motion‐driven electrical stimulation and microwave‐enhanced mild thermal therapy, enabling responsive curcumin delivery and scar‐reduced healing of infected diabetic wounds, Figure 8I [163]. Ultimately, electrical and magnetic fields enhance therapeutic strategies by introducing biophysical cues such as directional cell migration, mechanotransduction, and localized heating, which chemical release alone cannot achieve [103]. However, whether the benefits of these modalities outweigh the increased device complexity in clinical wound care requires direct comparison with simpler stimuli‐responsive systems [103, 104, 176].

### 4.2 3D Printed Phytochemical Hydrogels

3D printing helps turn a hydrogel dressing into a defined device. It controls shape, thickness, porosity, and internal channels. It also supports repeatable manufacturing across batches. These points are central in recent wound dressing reviews [184]. The clinical translation of natural phytochemicals is frequently hampered by their intrinsic limitations, including poor water solubility, rapid oxidative degradation, and low bioavailability. 3D printing transforms these bioactive compounds from passive additives into functional components of sophisticated engineered scaffolds [184]. By precisely manipulating the spatial distribution of bioinks, 3D printing not only accommodates the encapsulation of hydrophobic molecules but also enables the fabrication of biomimetic architectures that modulate the wound microenvironment [184]. Figure 9A schematically summarizes the multifaceted advantages of this manufacturing approach, encompassing geometric customization and the preservation of bioactivity [185].

**FIGURE 9:**
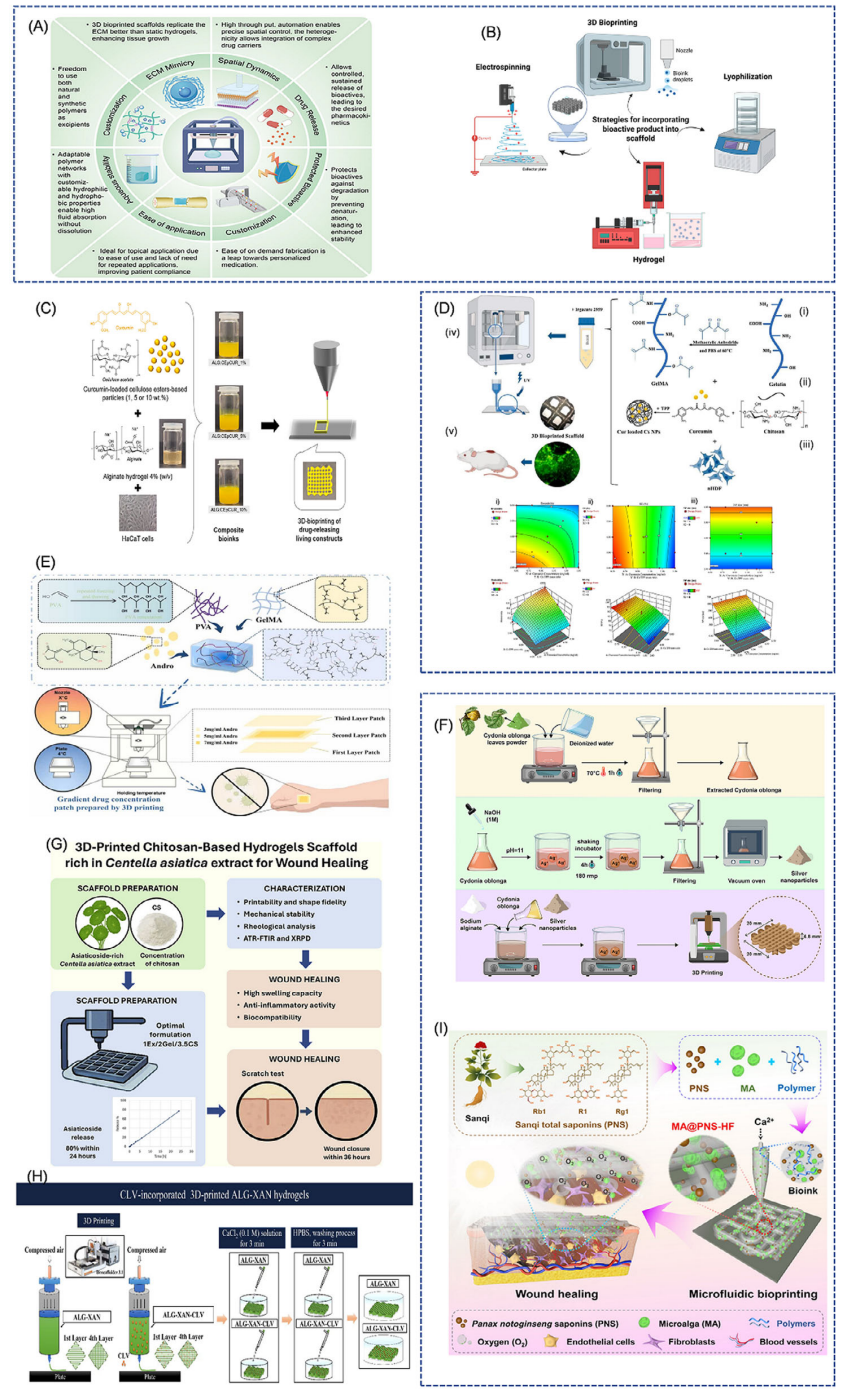
Engineering strategies and functional applications of 3D‐printed phytochemical‐loaded hydrogels in wound healing. A) Schematic illustration of the key features and biomedical advantages of 3D‐printed hydrogels. Reproduced with permission [185]. Copyright 2025, Wiley‐VCH GmbH. B) Common strategies for incorporating plant bioactive compounds into scaffold matrices. Reproduced under the terms of the CC‐BY 4.0 license [34]. Copyright 2025, The Authors, published by MDPI. C) Fabrication workflow for drug‐releasing living tissue analogs using alginate bioinks reinforced with curcumin‐loaded cellulose ester particles. Reproduced with permission [186]. Copyright 2023, American Chemical Society. D) Optimization and characterization of gelatin methacryloyl hydrogels incorporating curcumin‐loaded chitosan nanoparticles. Reproduced with permission [187]. Copyright 2024, Elsevier. E) Design of a multilayer GelMA‐PVA dual‐network hydrogel featuring a gradient distribution of Andrographolide for stage‐specific wound management. Reproduced with permission [188]. Copyright 2025, Elsevier. F) Schematic preparation of a nanocomposite dressing combining Cydonia oblonga extract with in situ synthesized silver nanoparticles. Reproduced with permission [189]. Copyright 2024, Elsevier. G) Formulation optimization of chitosan‐based scaffolds enriched with Centella asiatica extract to achieve enhanced printability and mechanical stability. Reproduced with permission [190]. Copyright 2025, Elsevier. H) Schematic of the extrusion‐based printing process utilizing alginate‐xanthan gum composite bioinks. Reproduced with permission [191]. Copyright 2023, American Chemical Society. I) Microfluidic bioprinting of “living” scaffolds co‐encapsulating Panax notoginseng saponins and oxygen‐generating microalgae. Reproduced with permission [192]. Copyright 2023, Science and Technology Review Publishing House.

To effectively harness these benefits, researchers employ various strategies to integrate phytochemicals into the hydrogel matrix, primarily dictating the release kinetics and mechanical stability, Figure 9B [34]. A critical advantage of integrating phytochemicals into 3D printed networks is the modulation of bioink rheology to enhance printability and shape fidelity. For instance, the incorporation of hydrophobic polyphenols often requires carrier systems that coincidentally improve the shear‐thinning behavior of the hydrogel. In a study addressing the poor solubility of curcumin, cellulose ester‐based particles were engineered to encapsulate the drug before dispersion into an alginate matrix. This strategy did not merely protect the bioactive payload; the inclusion of particles significantly increased zero‐shear viscosity and improved the resolution of the printed lattice, facilitating the stable fabrication of tissue analogs laden with keratinocytes, Figure 9C [186]. Similarly, utilizing a double‐entrapment strategy, chitosan nanoparticles loaded with curcumin were embedded within a GelMA hydrogel. This hierarchical design created a scaffold with highly interconnected porosity, essential for nutrient transport, while simultaneously exerting potent antibacterial activity against S. aureus and E. coli, Figure 9D [187].

Beyond structural reinforcement, 3D printing allows for the construction of heterogeneous scaffolds that mimic the graded complexity of native tissue, a feature unattainable by traditional casting methods. Addressing deep, infected wounds requires varying therapeutic concentrations at different tissue depths. A multi‐layered GelMA‐PVA dual‐network hydrogel was recently developed to deliver andrographolide in a gradient fashion. By printing layers with increasing drug concentrations, the scaffold achieved localized, high‐intensity antibacterial action in the deep wound bed while maintaining a cell‐friendly interface at the surface, effectively accelerating healing in full‐thickness defects, Figure 9E [188].

Furthermore, the versatility of extrusion‐based printing facilitates the use of “green” synthesized nanocomposites and multi‐functional blends without toxic crosslinkers. The combination of alginate with Cydonia oblonga seed extract, used to reduce silver ions into silver nanoparticles in situ, yielded a dressing with synergistic antimicrobial and antioxidant profiles. The 3D printed construct provided a moist environment that favored autolytic debridement, crucial for necrotic burn wounds, Figure 9F [189]. Similarly, the incorporation of Centella asiatica extract into chitosan‐based hydrogels has proven effective in modulating the enzymatic wound environment; by inhibiting hyaluronidase activity, these scaffolds prevented matrix degradation and supported fibroblast migration, Figure 9G [190]. In another approach tackling the printability of alginate, the inclusion of xanthan gum allowed for the stable extrusion of clove essential oil‐loaded scaffolds. This formulation leveraged the essential oil not just as an antimicrobial agent, but as a modulator of macrophage polarization, significantly downregulating nitric oxide production to mitigate chronic inflammation, Figure 9H [191].

The next frontier in this domain involves the convergence of phytochemical delivery with living therapeutic materials. Moving beyond static drug release, recent work has demonstrated a “living” scaffold comprising Panax notoginseng saponins (PNS) and autotrophic microalgae printed via microfluidic technology. In this symbiotic system, the PNS promotes angiogenesis while the microalgae continuously generate oxygen via photosynthesis, directly alleviating the hypoxic state typical of diabetic wounds. This dynamic interplay significantly enhanced collagen deposition and re‐epithelialization, marking a shift toward metabolically active dressings, Figure 9I [192]. Collectively, these advances illustrate that 3D printing is not merely a fabrication tool but a necessary enabler for unlocking the full therapeutic potential of phytochemicals in regenerative medicine [34, 185].

Despite these advances, 3D‐printed phytochemical hydrogels face key challenges for clinical translation. First, bioink stability remains a bottleneck: natural polymers including alginate and GelMA often require post‐printing crosslinking (such as Ca^2^+ treatment for alginate), a step that may degrade heat‐sensitive phytochemicals (including essential oils) or reduce viability of encapsulated cells [191, 193]. Photo‐crosslinking strategies pose an analogous risk: ultraviolet or blue‐light initiation generates free radicals and localized heat that can oxidize redox‐sensitive polyphenols such as curcumin and quercetin, eroding their antioxidant capacity before the dressing is ever applied. A second, frequently overlooked source of variability is incomplete fusion between adjacent printed filaments. In extrusion‐based printing, weak interfilament bonding leaves microscopic voids and density gradients across the construct, which translate into spatially heterogeneous swelling and uneven, poorly predictable local release of the encapsulated phytochemical. Second, printing speed is insufficient for large‐scale clinical use; current extrusion‐based systems typically require hours to print a single wound dressing, limiting their applicability in emergency settings where rapid treatment is critical [194]. Third, standardization of printing parameters (including pressure, temperature, and exposure time) across different phytochemical classes is lacking: parameters optimized for poorly water‐soluble phytochemicals like curcumin may not be suitable for hydrophobic terpenoids such as asiaticoside, leading to inconsistent phytochemical release kinetics and variable healing outcomes.

Future directions should focus on addressing these limitations through three targeted strategies: (1) Development of “self‐healing bioinks”, in which reversible dynamic covalent and supramolecular bonds are pre‐formed within the ink itself, so that gelation no longer depends on a separate post‐printing curing step and sensitive phytochemicals are spared the free‐radical, thermal and ionic insults of conventional UV or Ca^2^^+^ curing [125, 146]; alongside low‐temperature and visible‐light crosslinking chemistries (e.g., riboflavin‐ or ruthenium‐based visible‐light systems) that achieve gelation under mild, phytochemical‐friendly conditions; (2) Integration of AI to optimize printing parameters (including layer thickness and phytochemical loading concentration) for different wound types, AI models can predict hydrogel mechanical properties and phytochemical release profiles, reducing reliance on time‐consuming experimental trial‐and‐error; (3) Advancement of in situ 3D printing technologies (such as handheld printers) that can directly print hydrogels onto irregular wound surfaces, further enhancing personalization and improving practicality in clinical settings [194]. It should be emphasized, however, that self‐healing bioinks relocate rather than abolish crosslinking, and their benefit is conditional: because purely dynamic networks are viscoelastic and prone to creep, many reported self‐healing inks still incorporate a secondary permanent crosslink to secure shape fidelity and long‐term stability, thereby reintroducing the very processing step they are intended to avoid. The mechanical demands of a skin dressing are comparatively modest, so a fully dynamic formulation may prove sufficient in this specific context, but this has yet to be demonstrated for phytochemical‐loaded inks and remains an open question. More broadly, the challenges identified throughout this section, including the vast formulation design space, the difficulty of standardizing printing parameters across phytochemical classes, and the need for real‐time wound monitoring to guide adaptive therapy, collectively point to the need for computational approaches that can accelerate optimization and enable closed‐loop decision‐making. The following section examines how artificial intelligence is beginning to address these needs.

## 5 Artificial Intelligence in the Design and Optimization of Phytochemical Hydrogels

As discussed in Sections 3 and 4, the rational design of phytochemical hydrogel systems confronts several intertwined challenges: the vast combinatorial space of multi‐component botanical formulations, the complex and often non‐linear relationships between phytochemical loading and hydrogel network behavior, and the difficulty of simultaneously optimizing stimuli‐responsive release kinetics, printability, and therapeutic efficacy through conventional trial‐and‐error approaches. Artificial intelligence offers a transformative strategy to navigate this complexity. During the application of artificial intelligence in material discovery, AI‐based digital wound assessment has already emerged as an efficient and objective tool for quantifying wound size, granulation, tissue composition, and healing trajectories. Deep‐learning segmentation, 3D reconstruction, and automated planimetry can greatly reduce observer bias and enhance the reproducibility of preclinical and clinical trials. Figure 10 summarizes representative AI‐driven platforms spanning wound imaging, biochemical sensing, multimodal diagnostics, wearable monitoring, and predictive modeling, highlighting how digital technologies are reshaping the way wound status is quantified before informing hydrogel design. These AI frameworks encompass automated wound classification and segmentation (Figure 10A) [195], next‐generation biochemical sensing through intelligent wearable devices (Figure 10B) [196], multimodal deep‐learning diagnostic systems (Figure 10C) [197], contactless smart bandages powered by neural networks (Figure 10D) [198], and predictive computational models for treatment monitoring (Figure 10E) [199]. These technologies not only standardize wound evaluation but also generate high‐quality phenotypic and biochemical datasets that are essential for training AI models in material discovery and hydrogel optimization. The convergence of artificial intelligence technologies with hydrogel materials science has opened new frontiers in the development of intelligent therapeutic systems for biomedical applications [200, 201, 202]. Recent advances in machine learning algorithms and computational modeling have demonstrated remarkable potential in predicting and optimizing hydrogel properties, significantly accelerating the design process from traditional empirical approaches to data‐driven methodologies [203, 204]. Simultaneously, the integration of natural phytochemicals into hydrogel matrices has gained considerable attention due to their inherent biocompatibility, diverse therapeutic properties, and sustainable sourcing [8, 205]. The synergistic combination of AI‐guided design principles with phytochemical‐loaded hydrogel systems points toward a more rational, patient‐specific approach to wound therapy, in which intelligent algorithms are expected to help prioritize promising formulations for defined therapeutic outcomes [206, 207, 208]. While still in its early stages, integrating computational tools with phytochemical delivery is beginning to shorten development cycles and reduce reliance on exhaustive empirical screening. This efficiency gain is particularly valuable given the inherent structural complexity of plant derived bioactive compounds [209]. Throughout this section we therefore distinguish between capabilities that have been experimentally demonstrated for hydrogel or phytochemical systems and those that remain conceptual extrapolations, since the two are easily conflated in a field advancing this rapidly.

**FIGURE 10:**
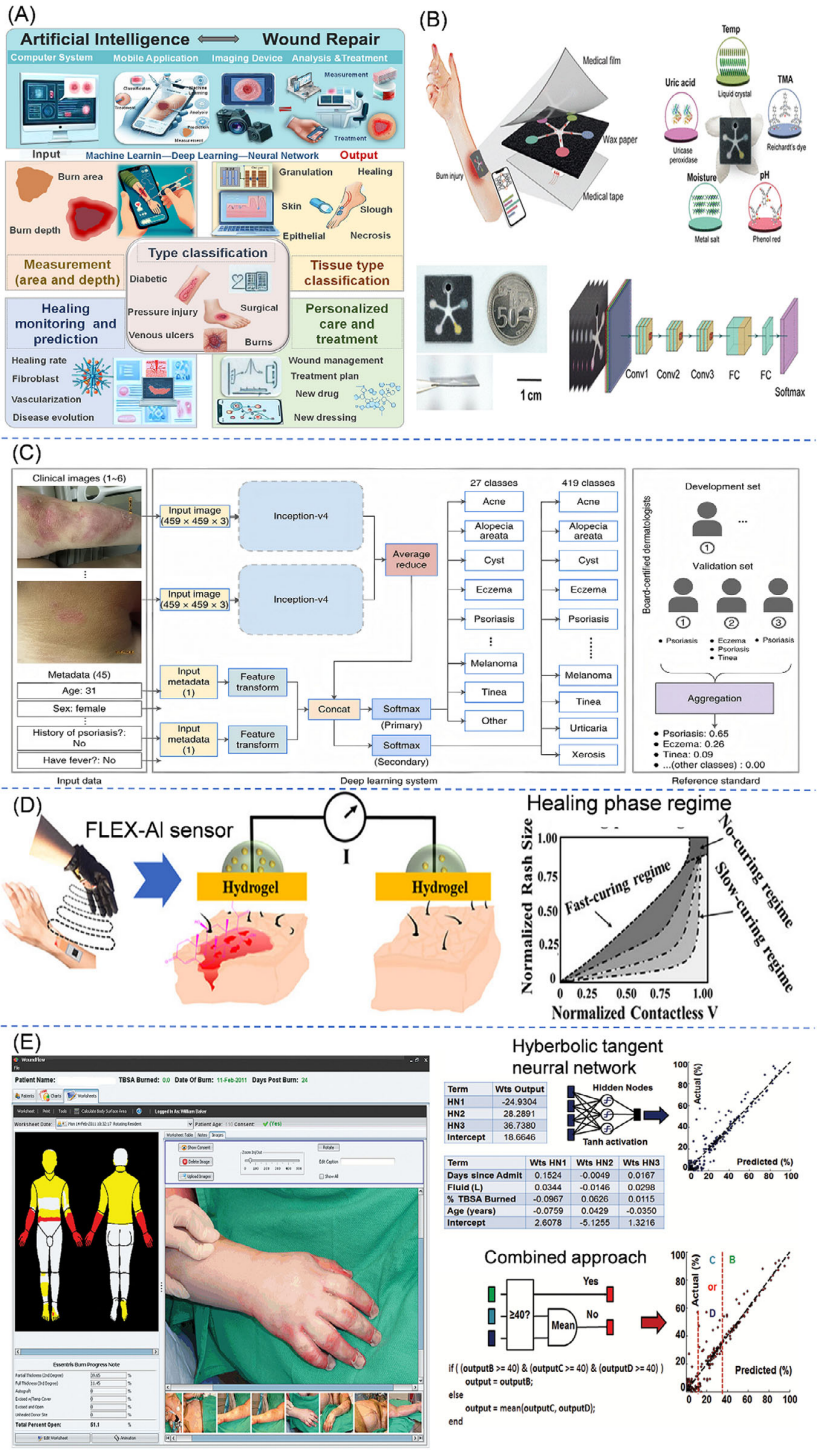
Artificial intelligence enabled technologies for wound assessment and decision support. A) AI‐assisted wound classification, segmentation, depth estimation, and healing prediction improve accuracy and reproducibility in clinical decision‐making. Reproduced with permission [195]. Copyright 2025, Ivyspring International Publisher. B) Battery free colorimetric wearable sensors integrated with machine‐learning algorithms enable real time biochemical monitoring of wound microenvironments. Reproduced with permission [196]. Copyright 2023, American Association for the Advancement of Science. C) Deep learning diagnostic systems incorporating multimodal clinical images and metadata provide high throughput and standardized wound characterization. Reproduced with permission [197]. Copyright 2020, Springer Nature. D) AI‐guided flexible sensors and smart bandages support non‐contact detection of wound states and dynamic healing trajectories. Reproduced with permission [198]. Copyright 2022, American Chemical Society. E) Digital wound documentation platforms and neural network based predictive models facilitate wound size evaluation and treatment response forecasting. Reproduced with permission [199]. Copyright 2018, American Burn Association.

### 5.1 Integration of Artificial Intelligence in Hydrogel Research

The conjunction of artificial intelligence and advanced hydrogel systems is fundamentally changing how therapeutic hydrogels are designed and optimized for complex physiological demands, such as chronic wound management [200, 210]. Traditional discovery processes for biomaterials, including hydrogels, are often constrained by time consuming, empirical methods, limiting the pace of innovation necessary to meet evolving biomedical needs [200, 211]. Machine learning, functioning as the core engine of this data‐driven revolution, accelerates the rational design of functionalized hydrogel formulations by extracting and decoding the intricate, non‐linear relationships governing the interplay between material composition, structure, and therapeutic function, Figure 11A [200, 212, 213]. This is particularly valuable for phytochemical hydrogels, where multi‐component botanical extracts can introduce complex, poorly parameterized interactions with polymeric networks and pronounced batch‐to‐batch variability. For instance, certain phytochemicals interact directly with the hydrogel matrix in ways that are difficult to anticipate from composition alone: quercetin perturbs gelation kinetics through host‐guest competition with the polymer network [52], and the redox‐active quinone shikonin exhibits a narrow safe‐to‐toxic concentration range [44]. Such compound‐specific effects on network formation and therapeutic window are poorly captured by first‐principles models, making them well suited to data‐driven optimization. Specifically, iterative workflows that couple computational modeling with experimental validation enable data‐driven prediction of gelation kinetics and rheological readouts (e.g., gel point/gel time, storage modulus evolution, viscosity and yield stress), accelerating formulation screening and process optimization, Figure 11B [214]. This capability is particularly relevant for phytochemical‐loaded systems, where poorly soluble or highly hydrophobic compounds may perturb network formation and printability, necessitating pre‐emptive formulation adjustment. This capability allows researchers to move beyond traditional trial‐and‐error approaches and engineer materials that more closely approximate the native ECM while possessing the requisite characteristics, such as high water content and biocompatibility, essential for tissue regeneration [200, 201].

**FIGURE 11:**
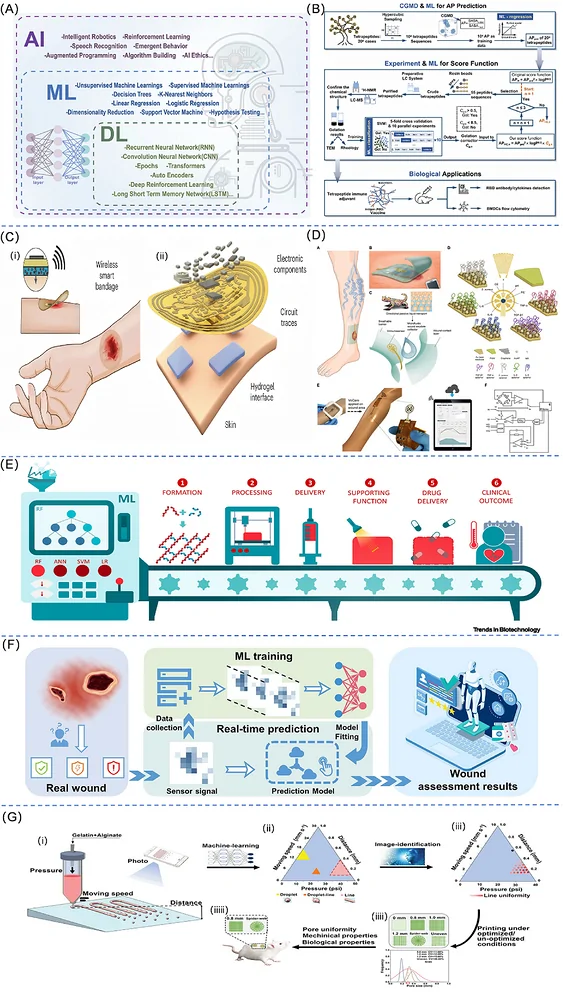
Artificial intelligence‐assisted design, fabrication, and functional optimization of phytochemical hydrogel systems: spanning three integrated stages: AI‐guided formulation design and property prediction, AI‐optimized fabrication, and AI‐enabled therapeutic monitoring and closed‐loop delivery. A) Hierarchical relationship between artificial intelligence, machine learning, and deep learning. Reproduced with permission [213]. Copyright 2025, Wiley‐VCH GmbH. B) Representative ML‐guided hydrogel discovery workflow integrating computational modeling, data generation, and iterative learning for predicting gelation‐related properties. Reproduced under the terms of the CC‐BY 4.0 license [214]. Copyright 2023, The Authors, published by Springer Nature. C) Schematic of a hydrogel‐integrated wireless smart bandage, illustrating the coupling of conductive hydrogel interfaces with flexible electronics. Reproduced with permission [216]. Copyright 2023, Springer Nature. D) Multiplexed hydrogel‐based sensing platform for continuous monitoring of wound‐relevant biochemical and physicochemical parameters. Reproduced with permission [219]. Copyright 2021, The American Association for the Advancement of Science. E) Overview of ML applications across key stages of hydrogel system development, including formulation prediction, printability optimization, and functional tuning. Reproduced with permission [211]. Copyright 2023, Elsevier. F) Data‐driven framework for intelligent wound assessment based on sensor‐enabled hydrogel systems and real‐time ML prediction. Reproduced with permission [218]. Copyright 2022, Elsevier. G) AI‐assisted high‐throughput optimization of extrusion‐based hydrogel printing parameters to achieve uniform and reproducible scaffold architectures. Reproduced with permission [208]. Copyright 2022, Wiley‐VCH GmbH.

The effective clinical translation of next‐generation hydrogels hinges upon rigorous optimization of their fundamental properties, including mechanical strength, degradation kinetics, and stimuli‐responsive release profiles of bioactive phytochemicals, a task uniquely suited to advanced ML architectures [200]. Sophisticated ML techniques, such as deep neural networks (DNNs) and support vector machines (SVMs), have been applied to predict these complex structure‐property relationships from initial design parameters, offering a route toward tailoring encapsulation efficiency and release duration to particular wound contexts, Figure 11E [201, 211, 215]. Furthermore, Figure 11G, this integration could help to address manufacturing bottlenecks by facilitating high‐throughput screening and optimization of fabrication parameters, such as those used in 3D bioprinting [208]. By predicting how structural inputs influence macroscopic properties, AI‐guided design could support the creation of customized, robust scaffolds with features such as enhanced adhesion and self‐healing capability, helping to keep the spatial distribution of phytochemicals stable under the mechanical stresses inherent in wearable and implantable applications [200, 211].

A key application envisaged for this synergy is intelligent wound management, particularly for chronic wounds that fail to heal and that demand real‐time surveillance and proactive intervention [211, 216]. Progress toward this goal is, however, uneven, and it is important to separate what has already been demonstrated from what remains a design concept.

The demonstrated foundation is presently electronic rather than algorithmic. Conductive hydrogel interfaces provide the low impedance and mechanical compliance required for robust signal transduction at the wound bed, Figure 11C [216, 217]. Built on such an interface, a battery‐free, wirelessly powered smart bandage has been shown to monitor skin impedance and temperature continuously and to deliver programmed electrical stimulation in response to the wound environment, with treated animals healing approximately 25% more rapidly and showing approximately 50% greater dermal remodeling than controls in murine wound models; the same device detected the early onset of infection and modulated stimulation accordingly [216]. Two features of that system deserve emphasis, because they are frequently blurred in discussions of intelligent dressings: its feedback loop is implemented in circuitry rather than in a trained predictive model, and its actuator delivers an electrical cue rather than a therapeutic payload. Machine learning has so far entered this space at the interpretive layer instead, where multiplexed sensing platforms and data‐driven models are used to classify wound status and to forecast healing trajectory from biochemical and physicochemical readouts, Figure 11D,F [210, 218, 219].

What has not yet been demonstrated, to our knowledge, is the final step of this vision: feedback‐regulated release of an encapsulated phytochemical payload governed by a predictive model. Extending the closed‐loop architecture from electrical to pharmacological actuation would, in principle, allow the timing and dose of anti‐inflammatory or antimicrobial phytochemicals to be matched to the evolving wound state, thereby limiting the toxicity risks associated with phytochemical overdose while maintaining effective local concentrations. We present this as a prospective direction rather than an established capability. Realizing it will require actuation modules capable of metering a defined phytochemical dose from within a degradable dressing, sensing channels that report on the payload itself rather than only on bulk tissue properties, and, ultimately, prospective evidence that feedback‐controlled dosing outperforms fixed‐schedule release.

If validated at scale, such closed‐loop systems could shift wound management from scheduled dressing changes to data‐guided, adaptive treatment, though the regulatory path for AI‐embedded medical devices and the scarcity of standardized clinical wound datasets are two practical barriers that the field must address before this vision becomes routine [210, 213]. Despite these promising advances, several limitations should be noted. The most pressing is data scarcity. In materials science, most experimental datasets are too small for conventional deep learning, and this problem is especially acute for hydrogel formulation research [220]. Individual studies in this field are typically designed to test a limited set of candidate formulations rather than to generate training data, and inconsistent or incomplete reporting, together with the rarity of raw data deposited in machine‐readable form, further impedes the aggregation of results across laboratories into the standardized datasets that data‐hungry models require [220, 221]. We are not aware of any systematic quantification of dataset size across the phytochemical hydrogel literature, and establishing such a baseline would itself be a useful contribution. Model interpretability is another barrier. In pharmaceutical development, complex deep learning models often function as “black boxes” whose decision logic cannot be easily traced back to physicochemical principles [222]. This is problematic because regulatory agencies, including the FDA and EMA, increasingly require that AI‐derived predictions in medical devices and pharmaceutical products be transparent and mechanistically justifiable [223]. For phytochemical hydrogel formulations, where composition directly affects patient safety, simpler and more interpretable models such as random forests or Bayesian optimization may be more appropriate in the near term, even if they sacrifice some predictive accuracy. Generalizability across phytochemical classes is also unproven. A model trained on polyphenol hydrogel data, for instance on catechol‐boronate ester bonding dynamics, cannot be assumed to work for alkaloid or terpenoid systems without substantial retraining. These compound families differ in hydrogen‐bonding capacity, charge state, and hydrophobicity, all of which alter how they interact with polymer networks [200]. Cross‐class transfer learning strategies that account for these structural differences remain largely unexplored in the phytochemical hydrogel literature. Finally, embedding sensors, wireless communication modules, and AI inference hardware into biodegradable wound dressings adds engineering complexity and manufacturing cost [210, 216]. Whether the clinical benefit of such intelligent systems justifies their cost, particularly in resource‐limited settings where chronic wounds are most prevalent, is a question that awaits formal health‐economic evaluation [2].

Molecular modeling approaches have significantly advanced modern hydrogel design by enabling accurate computational prototyping, multi‐scale material characterization, and systematic synthesis optimization for biomedical applications. Established simulation methods, including molecular dynamics [224, 225], density functional theory [226, 227], finite element analysis [228], and computational fluid dynamics, provide comprehensive insights into hydrogel behavior across atomic, molecular, and macroscopic scales [229, 230]. While powerful, the high computational cost and reliance on predefined physical models of these methods can limit their capacity for large‐scale screening and novel material discovery. These computational tools accelerate research progress in drug delivery systems, tissue engineering, and biosensing technologies. Artificial intelligence has emerged as a transformative force in hydrogel optimization, demonstrating remarkable capabilities in predicting material properties and guiding design decisions in selected model systems [200]. For example, Artificial neural networks have been used to predict discrete swelling states of temperature‐responsive PNIPAAm hydrogels from synthesis parameters, achieving a relative prediction error of 0.11 in the test dataset [231]. This approach enables researchers to rapidly screen optimal formulations without extensive experimental trials, significantly reducing development time and costs. Machine learning models have also been applied to drug release prediction from degradable hydrogels, where algorithms integrate time‐varying diffusivity and geometric data to accurately forecast release profiles [232]. For instance, multiscale diffusion models combining hydrodynamic, free volume, and obstruction theories have demonstrated superior performance in predicting solute transport through poly (ethylene glycol) hydrogels compared to traditional theoretical frameworks [233]. AI‐guided membrane design strategies have been extended to hydrogel systems, enabling property prediction, virtual screening, and autonomous optimization of material compositions [234]. These intelligent systems can simultaneously optimize multiple parameters, including mechanical strength, biodegradation rate, and drug loading capacity, through multi‐objective algorithms that would be computationally prohibitive using conventional approaches [235].

### 5.2 AI‐Modulated Natural Phytochemicals in Hydrogel Systems

The synergistic integration of artificial intelligence with natural product research has enabled recent advancements in the discovery and optimization of bioactive payloads for hydrogel systems. Unlike the structural engineering of the polymer matrix discussed in Section 5.1, the application of AI here focuses on orchestrating the selection and molecular modulation of plant‐derived therapeutic compounds [236, 237]. Machine learning‐based approaches have demonstrated exceptional proficiency in navigating the vast chemical space of natural products, enabling the strategic identification of high‐potency polyphenols, alkaloids, and terpenoids that serve as the functional core of bioactive dressings [238, 239].

Advanced computational models, including deep neural networks and transformer architectures, facilitate the critical “hit‐to‐lead” phase by predicting bioactivity and stability profiles before wet‐lab formulation begins. At the molecular level, the effectiveness of this optimization depends on information‐rich representations of natural product structures. Recent advances in sequence‐based deep learning models, together with machine learning‐driven virtual screening strategies, have substantially improved the identification of bioactive natural products from large libraries, Figure 12A,B [236, 240]. For instance, InflamNat achieved AUC values of 0.842 and 0.872 for predicting anti‐inflammatory activity and compound–target interactions, respectively [236], while disease‐specific deep‐learning workflows have also been used for candidate prioritization in other pathological contexts [241]. By resolving the complex distribution patterns of these phytochemicals, AI enables the rational design of delivery platforms in which payload bioavailability can be improved [237, 242].

**FIGURE 12:**
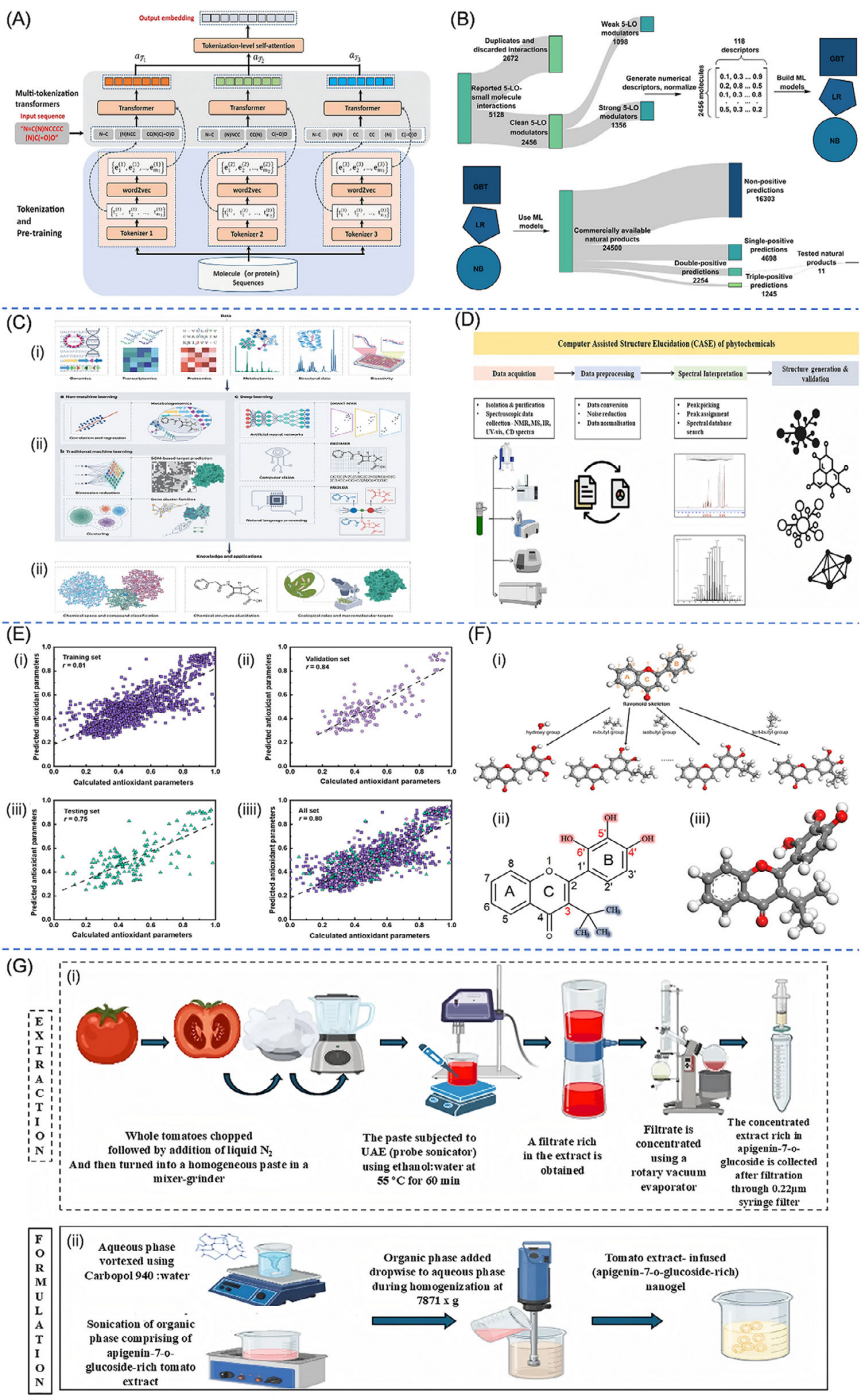
AI‐enabled discovery, optimization, and integration of natural phytochemicals for hydrogel‐based delivery systems. A) Multi‐tokenization transformer architecture for high‐quality representation learning of natural product molecular sequences in bioactivity prediction tasks. Reproduced under the terms of the CC‐BY 4.0 license [236]. Copyright 2022, The Authors, published by Springer Nature. B) ML‐based virtual screening workflow for identifying bioactive natural products targeting inflammatory pathways, exemplified by predictive modeling of 5‐lipoxygenase modulators. Reproduced with permission [240]. Copyright 2023, American Chemical Society. C) Applications of artificial intelligence in natural product and drug discovery. Reproduced with permission [247]. Copyright 2023, Springer Nature. D) Computer‐assisted structure elucidation framework for phytochemicals, combining spectroscopic data preprocessing, spectral interpretation, and structure generation. Reproduced with permission [207]. Copyright 2025, Springer Nature. E) Correlation analysis between ML‐predicted and calculated antioxidant parameters, demonstrating model performance across training, validation, and test datasets. Reproduced with permission [248]. Copyright 2024, American Chemical Society. F) Machine learning‐guided molecular design of flavonoid derivatives, illustrating structure‐activity relationships and three‐dimensional structural optimization. Reproduced with permission [248]. Copyright 2024, American Chemical Society. G) AI‐assisted optimization of green extraction and formulation processes for phytochemical‐loaded hydrogels, exemplified by ultrasonication‐assisted extraction and hydrogel incorporation of apigenin‐7‐O‐glucoside. Reproduced with permission [101]. Copyright 2025, Springer Nature.

Beyond individual prediction tasks, artificial intelligence now enables an integrated “discovery‐to‐design” workflow for natural phytochemicals, encompassing compound prioritization, structural elucidation, and rational molecular optimization [240]. Specifically, machine learning algorithms allow for the automated deconvolution of complex botanical extracts, a traditional bottleneck in phytochemical research. For instance, Elastic Net and Random Forest algorithms have been successfully employed to identify xanthohumol as a primary anti‐inflammatory agent from hop extracts (Humulus lupulus) by analyzing mass spectrometry data, validating biological activity without extensive bioassay‐guided fractionation [243].

Moving from discovery to modification, deep recurrent neural networks, particularly the quasi‐biogenic molecule generator, have substantially expanded the accessible design space. These models generate virtual compound libraries that maintain natural product characteristics while systematically optimizing physicochemical properties (e.g., solubility and lipophilicity) to ensure compatibility with hydrogel matrices [244]. Furthermore, transformer‐based deep learning architectures have been successfully implemented to perform de novo design of natural‐product‐inspired modulators, generating synthetic mimetics that retain the bioactivity of their natural templates while improving stability in aqueous environments [245]. Advanced bioactivity descriptors now enable the prediction of behavior for previously uncharacterized compounds, with signature‐activity relationship models achieving substantial performance improvements over traditional classifiers in forecasting membrane permeability and therapeutic efficacy [246]. Such AI‐enabled pipelines collectively accelerate the transition from raw plant material to optimized molecular payload, as illustrated by integrated workflows for compound screening, structure elucidation, and structure activity‐guided design, Figure 12C–F [207, 247, 248].

Building upon these molecular advances, artificial intelligence also serves as a crucial mediator in bridging phytochemicals with wound healing mechanisms. Comprehensive AI‐driven analyses have identified key pharmacological targets, including the suppression of inflammatory cytokine cascades and the modulation of antioxidative enzyme activity in plant extract‐loaded formulations [249]. Notably, neural network models with cross‐validation architectures have been used to predict the ADMET/toxicity profile of β‐caryophyllene, with endpoint‐specific accuracy values, some of which approached 0.84 [250].

Finally, at the processing level, AI extends beyond molecular design to optimize upstream extraction and incorporation strategies. Machine learning approaches have been applied to determine optimal “green” extraction parameters (e.g., solvent ratios, temperature) to maximize yield and batch‐to‐batch consistency, a critical prerequisite for reproducible hydrogel fabrication [251, 252]. Representative examples include machine learning‐assisted optimization of extraction processes and the subsequent consistent incorporation of these phytochemical‐rich extracts into advanced hydrogel matrices, Figure 12G [101]. Despite this rapid progress, it is important to delineate the current limits of the field. Current AI applications remain fragmented across isolated tasks, including machine‐learning‐based evaluation of natural products [238], formulation and gelation optimization [214, 217], extraction‐process optimization [251, 252], and printing‐fidelity optimization [211]. Integrated pipelines that jointly optimize phytochemical selection, hydrogel formulation, release behavior, and fabrication remain rare. The closest existing examples are confined to narrow sub‐problems, such as machine‐learning‐assisted optimization of essential‐oil and flavonoid extraction from Citrus aurantium [251] and AI‐assisted optimization of green extraction coupled to downstream hydrogel incorporation [101]. Bridging these isolated modules into an end‐to‐end, multi‐objective optimization framework therefore represents the principal open challenge, and the most promising near‐term opportunity, for the phytochemical‐hydrogel field.

## 6 The Clinical Application Prospects of Natural Phytochemicals‐Based Hydrogels

Preclinical investigations demonstrate that natural phytochemical hydrogels possess favorable biocompatibility and accelerate wound repair in animal models by conferring multiple therapeutic functions [253]. However, progressing beyond in vitro cell cultures and in vivo studies, the rigorous clinical implementation of this technology is imperative. Clinical trials validate essential information, including treatment effectiveness, cost, safety profile, and inherent risks. This clinical assessment determines the true potential of phytochemical hydrogels in wound management, facilitating technology transfer from the laboratory to patient care. Meta‐analyses aggregating data across several studies confirm that combination herbal hydrogel therapy significantly increases the likelihood of complete wound healing by 70% compared to conventional care (RR = 1.70) [254]. This efficacy is matched by strong safety profiles. Specifically, a pilot randomized controlled trial demonstrated that a nano‐hydrogel integrating Quercetin (Que) and Oleic Acid (OA) achieved total re‐epithelialization in 92.8% of diabetic patients within 60 days, significantly outpacing the hyaluronic acid (HA) control group (57.1%) [255]. Furthermore, the Que/OA formulation reduced average healing time and, critically, showed zero incidence of local infection, unlike the HA control group where 57.2% developed infection [255]. This initial clinical validation suggests that synergistic phytochemical combinations offer an effective and safe topical approach for complex diabetic skin repair. Similarly, in a prospective randomized double‐blind pilot trial in neuropathic diabetic foot ulcers (n = 50), a topical dressing containing 0.1% P1G10 (a plant latex‐derived proteolytic fraction) achieved a higher complete‐healing incidence than hydrogel standard care, with representative clinical images illustrating ulcer closure over 110 days, Figure 13A [256].

**FIGURE 13:**
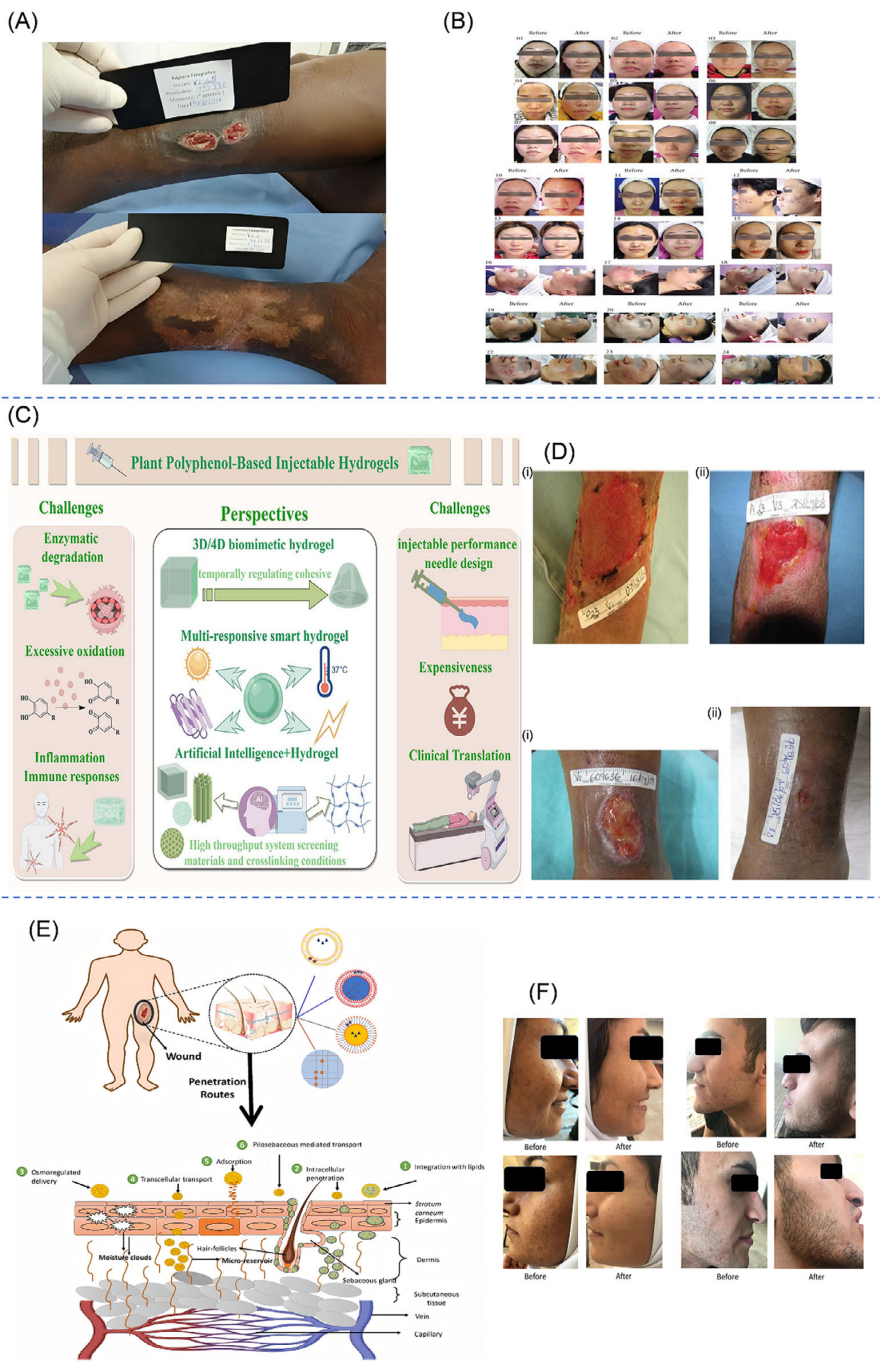
Representative clinical outcomes and a translational framework for natural phytochemical‐based hydrogels, integrating wound/acne case images and delivery rationale. A) Baseline vs 110‐day neuropathic DFUs after 0.1% P1G10 dressing. Reproduced with permission [256]. Copyright 2018, Springer Nature. B) Baseline vs day‐14 images from 24 acne subjects treated with a multi‐component herbal hydrogel. Reproduced under the terms of the CC‐BY 4.0 license [260]. Copyright 2021, The Authors, published by Hindawi. C) Roadmap for plant polyphenol‐based injectable hydrogels emphasizing 3D/4D biomimicry, multi‐responsive smart designs, and AI‐assisted materials/crosslinking optimization. Reproduced with permission [9]. Copyright 2025, Wiley‐VCH GmbH. D) Before‐and‐after images of venous leg ulcers comparing MTC‐2G vs blank hydrogel, illustrating heterogeneity of clinical benefit. Reproduced with permission [263]. Copyright 2011, Blackwell Publishing Ltd and Medicalhelplines.com Inc. E) Nano‐drug delivery systems topical penetration routes (lipid integration, transcellular/intracellular transport, and pilosebaceous pathways) supporting enhanced local bioavailability. Reproduced with permission [271]. Copyright 2023, Springer Nature. F) Representative before‐after images following thymoquinone‐standardized Nigella sativa hydrogel therapy. Reproduced with permission [264]. Copyright 2020, John Wiley and Sons.

The efficacy of phytochemical combination hydrogels stems from a systems pharmacology approach, tackling the multifaceted pathophysiology of chronic wounds simultaneously [257]. For instance, Que (a flavonoid) and OA (a fatty acid) synergistically combine potent anti‐inflammatory and antioxidant activity with immune response modulation [258, 259], promoting tissue viscoelasticity and keratinocyte proliferation via the hyaluronic acid base [255]. Beyond DFUs, multi‐component hydrogels have shown promise in other challenging dermatological conditions. A complex herbal hydrogel containing green tea, ginger, Phyllanthus emblica, and salicylic acid successfully treated moderate‐to‐severe acne vulgaris in preliminary human trials, resulting in over 75% recovery in a significant portion of subjects within just 14 days, Figure 13B [260]. This effect relies on the combined action of anti‐inflammatory properties from tea polyphenols, antioxidative stress reduction from ginger, and keratolytic effects from salicylic acid, addressing microbial colonization and hyperkeratinization [261, 262]. Conversely, outcomes can be heterogeneous: an independent randomized comparative trial evaluating Mimosa tenuiflora cortex extract (MTC‐2G) hydrogel for venous leg ulcers showed no significant superiority over the blank hydrogel, although MTC‐2G successfully exhibited anti‐inflammatory effects by reducing neutrophilic infiltrate density, Figure 13D [263]. This discrepancy likely reflects differences in wound etiology, patient population, and phytochemical dose optimization. The quercetin/oleic‐acid study involved patients with chronic diabetic foot wounds, including DFUs unresponsive to standard treatment [255], whereas the MTC‐2G trial involved chronic venous leg ulcers [263]. The contrasting outcomes may reflect differences in wound etiology, patient population, formulation, and phytochemical dose, although the available studies do not allow the responsible factors to be distinguished conclusively. These contrasting outcomes highlight that the efficacy of phytochemical hydrogels is not universal but depends on the match between the phytochemical's mechanism of action, the specific wound pathology, and the rigor of formulation quality control. Notably, a randomized double‐blind controlled trial (n = 60) showed that a thymoquinone‐standardized Nigella sativa hydrogel applied twice daily for 60 days achieved a 78% mean reduction in Investigator's Global Assessment severity and a 63.49% reduction in Acne Disability Index, with no adverse events, as illustrated by representative patient outcomes, Figure 13F [264]. In a prospective, double‐blinded randomized controlled study in 65 patients with diabetic foot ulcers, a hydrogel containing botanical extract was non‐inferior to a widely used comparator hydrogel and showed superior pain reduction with a trend toward greater wound surface‐area reduction, supporting the translational promise of botanical/phytochemical hydrogels while underscoring the need for larger confirmatory trials [265].

Despite the clear therapeutic promise demonstrated across various preclinical models and initial human trials, widespread clinical translation requires addressing major translational and regulatory challenges. A critical barrier is the standardization of herbal components, as phytochemical content inherently varies based on plant source, processing, and extraction methods [255, 266, 267]. Reproducible efficacy demands establishing defined pharmaceutical marker compounds (such as curcuminoids or asiaticoside) for consistent quality control [267]. Beyond standardization, the regulatory classification of phytochemical hydrogel dressings itself remains uncertain. Unlike single‐entity small‐molecule drugs, these systems combine multi‐component botanical extracts with synthetic or semi‐synthetic polymer matrices, and in some cases incorporate stimuli‐responsive nanoparticles or conductive fillers. This compositional complexity makes it difficult to assign them to a single regulatory category. Under the FDA framework, product classification should first be determined from the constituent parts, intended use, and mechanisms of action. If a phytochemical hydrogel contains both drug and device constituent parts and meets the definition of a combination product, its lead‐center assignment is based on the primary mode of action [268]. In the European Union, qualification as a medicinal product or medical device should be assessed under the medical device–medicinal product borderline framework, considering the product's principal intended action and whether that action is achieved by pharmacological, immunological, or metabolic means [269]. For phytochemical hydrogels specifically, demonstrating batch‐to‐batch consistency is further complicated by the inherent variability of plant raw materials. Environmental conditions, including soil water and fertility, can alter the accumulation of secondary metabolites, while cultivation, harvest, and primary processing must be controlled to ensure consistent quality of herbal starting materials [270]. Botanical raw materials additionally carry contamination risks that are largely absent from fully synthetic systems: heavy metals (e.g., lead, cadmium, arsenic) taken up from soil, and residual pesticides or herbicides from cultivation, both of which are capped by pharmacopoeial limits and should be screened lot‐by‐lot to satisfy good manufacturing practice. Meeting good manufacturing practice requirements will therefore demand validated analytical fingerprinting methods, such as HPLC or LC‐MS profiling against defined marker compounds, applied at every stage from raw material intake to final product release.

The manufacturing cost of intelligent phytochemical hydrogel systems also deserves scrutiny. Current laboratory‐scale fabrication often involves multi‐step procedures: phytochemical extraction and purification, nanoparticle synthesis, hydrogel crosslinking, and in some cases 3D printing and sensor integration. The cumulative expense of multi‐step fabrication may limit accessibility in low‐ and middle‐income countries where chronic wounds impose the heaviest economic burden [1, 2]. Prospective cost‐effectiveness analyses comparing these advanced systems against conventional wound dressings are currently lacking for phytochemical hydrogel products, and this gap should be addressed before large‐scale clinical adoption can be justified. The inherently low cost of plant‐derived raw materials compared to recombinant proteins or synthetic peptides may partially offset this premium, but only if scalable extraction and formulation processes are established. Continuous manufacturing approaches, including roll‐to‐roll coating and automated extrusion‐based printing, offer potential routes to reduce per‐unit cost, though their compatibility with heat‐sensitive phytochemicals remains to be validated [184, 194].

In summary, four interlocking barriers currently gate the clinical translation of phytochemical hydrogels, mirroring the roadmap in Figure 13C: (i) raw‐material standardization: controlling marker‐compound content, heavy‐metal and pesticide residues, and batch‐to‐batch variability; (ii) regulatory ambiguity: the uncertain drug/device/combination classification of multi‐component systems under FDA and EMA frameworks; (iii) manufacturing scalability and GMP compliance: translating multi‐step, often hand crafted laboratory protocols into validated, reproducible production; and (iv) health‐economic uncertainty: the current absence of formal cost‐effectiveness analyses, which is especially consequential for the low and middle income settings that bear the greatest chronic wound burden. Looking ahead, the field is moving toward developing sophisticated next‐generation hydrogels capable of personalized therapeutic delivery. These innovations include stimuli‐responsive systems that can dynamically react to the wound microenvironment, such as infection status, pH changes, or glucose levels prevalent in diabetic wounds. Given the low cost and high accessibility of natural phytochemicals, advancing these tailored hydrogel interventions holds immense potential for effective and sustainable chronic wound care, significantly alleviating the global economic burden of these complex diseases. Moreover, coupling phytochemicals with nano‐drug delivery systems and integrating them into hydrogel matrices may further improve topical bioavailability by lipid integration, intracellular/transcellular, and pilosebaceous pathways, Figure 13E [271, 272]. Consistent with recent forward‐looking analyses of plant polyphenol‐based injectable hydrogels, three translational priorities are emerging: (i) 3D/4D biomimetic architectures to improve biosafety while mitigating inflammation and immune activation, (ii) multi‐responsive smart networks enabling adaptive, on‐demand release, and (iii) AI‐assisted screening of hydrogel matrices and crosslinking conditions to accelerate formulation development, Figure 13C [9].

Looking further ahead, three emerging paradigms are likely to reshape phytochemical hydrogel development. First, multi‐omics‐guided phytochemical selection, which integrates wound‐tissue transcriptomic, proteomic, and metabolomic signatures with compound target databases, could replace empirical compound choice with patient and pathology matched payload design. Second, foundation models for biomaterials, large architectures pre‐trained on heterogeneous chemical, structural, and biological data, may enable zero‐ or few‐shot prediction of formulation behavior across phytochemical classes, mitigating the data‐scarcity and cross‐class generalizability limitations discussed in Section 5.1. Third, autonomous materials development platforms that couple AI‐directed experimental planning with robotic synthesis and high‐throughput characterization could close the design, make, test, and learn loop, compressing the optimization of multi‐component phytochemical hydrogels from years to weeks.

## 7 Conclusions

The integration of natural phytochemicals into advanced hydrogel matrices marks a significant advance in chronic wound management, combining the multi‐target bioactivity of botanical agents with the tunable properties of responsive hydrogel platforms. The development of stimuli‐responsive systems (sensitive to pH, ROS, and glucose) and 3D‐printed scaffolds has helped overcome key limitations of phytochemicals, including poor stability and low bioavailability, by enabling localized and temporally controlled release.

AI‐based methods are increasingly being applied to move formulation design from trial‐and‐error toward data‐driven optimization. Machine learning models can now predict structure‐property relationships and assist in formulation screening, reducing the number of experimental iterations required. However, clinical translation still faces unresolved challenges, including botanical standardization, batch consistency, and the regulatory classification of multi‐component hydrogel systems. Future work should prioritize validated analytical fingerprinting for quality control and larger, well‐controlled clinical trials across different wound types. However, current clinical evidence remains largely limited to small sample pilot trials. Progress on these fronts will determine whether phytochemical hydrogels can advance from early results into routine wound care.

## Author Contributions

**Yitao Zhou**: Conceptualization, Methodology, Investigation, Data curation, Writing – original draft, Visualization. **Xing Liu**: Conceptualization, Methodology, Investigation, Data curation, Writing – original draft, Visualization. **Cuiyi Wang**: Investigation, Data curation, Writing – original draft. **Xiaoxuan Zhao**: Investigation, Validation, Writing – original draft. **Pengrun Zhang**: Validation, Writing – review & editing. **Jingjing Gu**: Visualization, Writing – review & editing. **Yeqin Yang**: Conceptualization, Supervision, Writing – review & editing. **Malcolm Xing**: Conceptualization, Supervision, Writing – review & editing. **Qiuhua Sun**: Conceptualization, Funding acquisition, Project administration, Supervision, Writing – review & editing.

## Ethics Approval and Consent to Participate

Not applicable. This article is a comprehensive review of previously published literature and does not involve any new experimental studies on human participants or animal subjects performed by any of the authors. Therefore, ethical approval and informed consent were not required.

## Conflicts of Interest

The authors declare no conflicts of interest.

## Data Availability

Data sharing not applicable to this article, as no datasets were generated or analyzed during the current study.
